# Investigating the dynamics and uncertainties in portfolio optimization using the Fourier-Millen transform

**DOI:** 10.1371/journal.pone.0321204

**Published:** 2025-06-17

**Authors:** Muhammad Hilal Alkhudaydi, Aiedh Mrisi Alharthi

**Affiliations:** 1 Department of Mathematics and Statistics, College of Science, Taif University, Taif City, Saudi Arabia; 2 Department of Mathematics, Turabah University College, Taif University, Taif City, Saudi Arabia; University of Almeria: Universidad de Almeria, SPAIN

## Abstract

Many investors and financial managers view portfolio optimisation as a critical step in the management and selection processes. This is due to the fact that a portfolio fundamentally comprises a collection of uncertain securities, such as equities. For this reason, having a solid understanding of the elements responsible for these uncertainties is absolutely necessary. Investors will always look for a portfolio that can handle the required amount of risk while still producing the desired level of expected returns. This article uses feature-based models to investigate the primary elements that contribute to the optimal composition of a specific portfolio. These models make use of physical analyses, such as the Fourier transform, wavelet transforms and the Fourier–Mellin transform. Motivated by their use in medical analysis and detection, the purpose of this research was to analyse the efficacy of these methods in establishing the primary factors that go into optimising a particular portfolio. These geometric features are input into artificial neural networks, including convolutional and recurrent networks. These are then compared with other algorithms, such as vector autoregression, in portfolio optimisation tests. By testing these models on real-world data obtained from the US stock market, we were able to obtain preliminary findings on their utility.

## Introduction

The overarching goal of this research is to identify the most effective strategy for portfolio management. To achieve this, we employed various methods for extracting information to identify the most effective components for portfolio optimisation.

Portfolio optimisation is a key component of contemporary finance, as it helps investors maximise profits while lowering risk. Due to rapid improvements in technology and the increasing complexity of financial markets, there is a growing need for sophisticated strategies to efficiently optimise portfolios [1]. This study seeks to provide a thorough understanding of cutting-edge approaches and their applications to portfolio optimisation using MATLAB. An outline of the study is first presented. Then, a section on the theory explores the guiding concepts of each of the seven topics covered.

The study goes on to compare the outcomes of three portfolio optimisation strategies: vector autoregression (VAR) (as a baseline), continuous wavelet transform (CWT) combined with a convolutional neural network (CNN), and the Fourier–Mellin transform (FMT) combined with a recurrent neural network (RNN). The purpose of the paper to determine the best method for optimising portfolios under various market scenarios by contrasting the performance of several feature extraction methods based on deep learning.

The application of machine learning methods, including automated machine learning (AutoML), CNNs, and long short-term memory (LSTM) networks, has garnered considerable interest within the dynamic field of investment strategies. The use of these methodologies, which are frequently combined with traditional statistical approaches such as VAR, CWT, and the FMT, presents opportunities for improving investment decision-making and portfolio management.

Portfolio optimisation is the process of choosing the best feasible set of assets to accomplish a particular investment goal, while taking the investor’s risk tolerance into account [2]. Modern portfolio theory (MPT), which established the concepts of diversification and the efficient frontier, serves as the theoretical underpinning of portfolio optimisation [3]. To reduce risk, diversification includes spreading investments across a variety of assets, while the efficient frontier is the set of ideal portfolios that provide the best expected return for a given level of risk. The Black–Litterman model, downside risk measures, and resilient optimisation techniques are only a few of the new optimisation techniques and risk measures that have been introduced as a result of the continued development and extension of MPT by numerous studies [4].

A study by Feng *et al*. conducted an in-depth examination of the contributions of different frequency components to image characteristics using weighted standard deviation (WSD) and weighted column standard deviation-based filtering to investigate image features [5]. The suggested methodology employs a weighted evaluation, which highlights specific aspects based on their importance and provides a numerical measure for quantitative analysis. Although WSD is computationally complex and requires careful interpretation, its ability to detect both high- and mid-frequency components allows for a comprehensive understanding of image quality [5]. The authors use wavelet spectral density to build a strong statistical model that accurately shows how different frequency components affect each other. This could make the model useful for processing and analysing images [5].

Although the work introduces advanced approaches for image registration and analysis using WSD methodologies, it also directly addresses the influence of noise as a key limitation, especially in short-exposure images. For rotational and scaling shifts, the authors agree that noise may reduce the accuracy of relative shift calculations. In this regard, the proposed FMT approach may not be as effective. Robust noise reduction methods must be used to address this issue and achieve improved outcomes from image processing.

The goal of portfolio optimisation is to provide investors with guidance for controlling their portfolios by determining the optimal allocations in different markets. Ali *et al*. analysed different portfolios by experimenting with changes in optimal weights for two eras – pre-COVID and during the COVID pandemic – in Asia Pacific [6]. They implemented various models as a core feature of their study, including dynamic conditional correlation (DCC), which is an extension of constant correlation estimation; multivariate and bivariate DCC-generalised autoregressive conditional heteroskedasticity (GARCH) models; the standard GARCH (1,1) model; and portfolio optimisation methods such as mean-variance (MV) and safe-haven dynamics, to provide protection during market conditions (a hedge) [6].

Additionally, the use of DCC-GARCH models was investigated to study green and non-green cryptocurrencies, diversification, risk management, and the impact of green assets on various equity portfolios by analysing risk–return dynamics [7]. This study evaluated the effectiveness of several portfolio optimisation techniques and weight constraints on assets to gain a deeper understanding of their practical implementations. By testing the safe-haven dynamics of specific cryptocurrencies, the study offers valuable insights into cross-market hedging and safe-haven opportunities for investors during periods of extremely low returns [7]. The assumptions used in the study, including GARCH modelling, which fails to capture certain market dynamics (such as jumps), may impact its robustness. Methods used to determine the optimal weights may also be significantly affected by the addition of factors such as liquidity limitations, investor preferences, and transaction costs, among others. Therefore, combining these studies with deep learning methods is important to investigate the key factors behind portfolio optimisation dynamics. Deep learning approaches have the power to handle various complexities, such as non-linear patterns in data, which is missing in traditional statistical models such as GARCH. Another advantage to deep learning methods is that they learn from data automatically, in contrast to the GARCH model, which depends more on manual feature engineering [8].

Artificial neural networks with many layers are the focus of the machine learning field known as deep learning. These networks can recognise subtle correlations and patterns in data [4], making them appropriate for a range of financial applications including portfolio optimisation [9]. Large datasets can be used to train deep learning models, enabling them to recognise complex patterns in financial data that may be challenging to find using more conventional techniques [10]. This may result in more accurate portfolio allocation decisions and better asset return estimates. Feedforward neural networks, CNNs, RNNs, and autoencoders are a few of the common deep learning architectures used in finance.

Specialised neural network architectures such as CNNs and RNNs are designed for particular kinds of data. CNNs have been used to evaluate financial time series data for a variety of tasks, including predicting stock prices and spotting market trends [11]. CNNs are particularly well suited for processing grid-like data, such as pictures or time series data. The temporal dependencies in financial time series data have been modelled using RNNs, which are built to process sequential data. Due to their capacity to recognise intricate patterns and correlations in financial data, both CNNs and RNNs have demonstrated promise in financial applications including portfolio optimisation. Popular RNN variants have been used to solve the vanishing gradient problem (which can occur in conventional RNNs during trainings), including LSTM and gated recurrent unit (GRU) networks.

By projecting high-dimensional data onto a lower-dimensional space while retaining as much of the data’s variance as possible, dimensionality reduction techniques seek to reduce the complexity of huge datasets. One common linear transformation method used to reduce dimensionality is principal component analysis (PCA) [12]. PCA can help uncover important characteristics influencing asset returns in the context of portfolio optimisation and help minimise the dimensionality of the optimisation issue [13]. This can in turn reduce the danger of overfitting and increase the computational efficiency of portfolio optimisation algorithms. Other dimensionality reduction methods have also been investigated for a variety of applications within finance, including t-distributed stochastic neighbour embedding and independent component analysis.

Multivariate extensions of autoregressive models known as VAR(p) models are capable of capturing the dynamic interactions between several financial variables. Important insights for portfolio management have been made by using these models to simulate the coupled dynamics of asset returns, interest rates, and other macroeconomic variables [14]. VAR(p) models can assist in increasing the accuracy of portfolio optimisation algorithms and improve investment decisions by incorporating data on many financial factors [15]. To address key difficulties in financial time series analysis, such as cointegration and model uncertainty, extensions of VAR models have been developed, such as the vector error correction model and the Bayesian vector autoregressive model.

A time series can be divided into multiple frequency components using a wavelet transform, which enables researchers to examine the data at various time scales [10]. This method has been used to capture both short- and long-term patterns in asset returns in financial time series data [15]. Wavelet transforms can facilitate increased precision in portfolio optimisation algorithms and result in better investment decisions by combining data from various time scales [16]. Numerous financial applications, including volatility forecasting, risk management, and market efficiency analysis, have used wavelet-based techniques.

The FMT combines the Fourier transform and polar coordinates to examine a signal’s frequency and scale content [17]. By using this combination, the FMT can examine the frequency and scale content of financial data, resulting in a more thorough understanding of the data [18]. The FMT has been used by researchers in a number of image processing applications, including classification problems, to showcase its ability to process and analyse data using deep learning methods [5].The FMT can assist in increasing the accuracy of portfolio optimisation algorithms, resulting in better investment decisions by using data from both the frequency and scale domains.

This study introduces feature-based models that contribute to the field of portfolio optimisation in the following ways:

1. Feature extraction is considered as a vital component for data analyses, with financial data being no exception. Investigating the underlying uncertainty of portfolio dynamics is a difficult task when relying only on historical prices or returns of assets, necessitating the use of a model able to learn from the data, such as a deep learning model.
2. The FMT has many applications in image processing, including registration and rotation. This works by extracting the important geometric features using the Fourier transform along with polar coordinates. In addition, the wavelet transform is a beneficial tool in financial time series analysis, because it can handle non-stationary financial data, whereas the Fourier transform only deals with stationary cases.
3. Integrating deep learning with the wavelet transform and FMT can lead to state-of-the-art feature-based deep neural network models capable of capturing the important elements of a financial portfolio. Our goal is not only to assess our models’ predictability but also to evaluate their ability to investigate the core data behind the portfolio dynamics.

This paper is divided into four sections. Section Mathematical explanation of tools used in this paper explains the mathematical methods necessary to build feature-based model blocks. These include VAR, PCA, CWT, RNNs, CNNs and the FMT. Section System design focuses on the design of three main feature-based models, from data selection to their structure. These models include the VAR automated machine learning investment strategy model (VAR(1)-AutoML), and the CWT investment strategy model (CWT-CNN), and the FMT recurrent neural network (FM-LSTM). Section Results evaluates the models’ performance, beginning with statistical analysis for the probability measures. Section Conclusions and future work discusses the conclusions of the study by highlighting its strengths and limitations.

## Mathematical explanation of tools used in this paper

The goal of this section is to describe the key mathematical concepts employed in this study.

### Vector autoregression

VAR is a statistical model widely used in econometrics and applied statistics. It is employed to analyse the linear relationships between several time series variables [19]. The VAR technique enables the modelling of each variable in a multivariate time series by considering its past values as well as the past values of other variables [19, 20].

Consider a multivariate time series $\left\{{𝐲}_{t}\right\}$, where each ${𝐲}_{t}$ is a *p* × 1 vector of *p* endogenous variables at time *t*:

$${𝐲}_{t}=[\begin{array}{c} y_{1t}\\ y_{2t}\\ \vdots \\ y_{pt} \end{array}].$$ (1)

A VAR model of order *p* (VAR(*p*)) can be represented as:

$${𝐲}_{t}=𝐜+{𝐀}_{1}{𝐲}_{t-1}+{𝐀}_{2}{𝐲}_{t-2}+\ldots +{𝐀}_{p}{𝐲}_{t-p}+{𝐮}_{t},$$ (2)

where:

- **c** is a p×1 vector of constants (intercepts),
- ${𝐀}_{i}$ (for i=1,2,…,p) are p×p coefficient matrices, and
- ${𝐮}_{t}$ is a p×1 vector of error terms.

The error terms ${𝐮}_{t}$ are considered to have the following additional characteristics:

- The expected value of ${𝐮}_{t}$ is **0** ($E({𝐮}_{t})=0$), indicating it has a mean of zero.
- The expected value of ${𝐮}_{t}{𝐮}_{t}^{\prime }$ is Σ ($E({𝐮}_{t}{𝐮}_{t}^{\prime })=\Sigma$), where Σ is a positive definite matrix of size *p* × *p* that represents the covariance matrix of the error terms.
- The expected value of ${𝐮}_{t}{𝐮}_{s}^{\prime }$ is **0** ($E({𝐮}_{t}{𝐮}_{s}^{\prime })=0$) for t≠s, ensuring that the errors are not correlated with each other over time [19–21].

VAR models are used for predicting the behaviour of a set of variables,

performing impulse response analysis to investigate the impact of shocks,

and conducting variance decomposition to analyse the individual impact of each variable on the predicted errors [22].

$${𝐲}_{t}=𝐜+{𝐀}_{1}{𝐲}_{t-1}+{𝐮}_{t},$$ (3)

is the formula for the VAR(1) model.

The given equation states that **c** is a vector of constants, ${𝐀}_{1}$ is a matrix of coefficients, and ${𝐮}_{t}$ is a vector of error terms [19–21].

Based on the assumptions made by the model, the error terms ${𝐮}_{t}$ are assumed to meet certain criteria:

- **Zero mean:** $E[{𝐮}_{t}]=0$.
- **Constant covariance:** $E[{𝐮}_{t}{𝐮}_{t}^{\prime }]=\Sigma$.
- **No serial correlation:** $E[{𝐮}_{t}{𝐮}_{s}^{\prime }]=0$ for all t≠s.

Ordinary least squares (OLS) regression can be used to approximated the parameters 𝐜 and ${𝐀}_{1}$ [23]. To obtain the estimates, we run a regression on all the variables using their lagged values.

For the VAR(1) model to be stable, each eigenvalue of ${𝐀}_{1}$ must have a modulus smaller than one. The way the system reacts to shocks is set by the characteristic equation obtained from ${𝐀}_{1}$.

One way to observe the long-term effects of a single variable shock in a VAR(1) model is through the impulse response functions. The answers are obtained by raising ${𝐀}_{1}$ to an appropriate power.

Econometricians and finance econometricians frequently employ VAR models when studying the fluctuating connections between different time series. Financial market analysts also often use VAR models to track asset returns so as to analyse interactions and predict future moves [19–21].

VAR models are widely employed in the fields of econometrics and finance econometrics to examine the dynamic connections among numerous time series. They are commonly used in financial markets to analyse the log returns of assets, with the aim of understanding their relationships and making predictions about future movements.

Logarithmic returns are commonly employed in finance because of their advantageous characteristics, including time additivity and improved management of the multiplicative nature of asset values. The log return of asset *i* from time *t* − 1 to *t* for a given collection of assets is calculated using the formula:

$$r_{i,t}=\log\left(\frac{P_{i,t}}{P_{i,t-1}}\right).$$ (4)

The symbol *P*_*i*,*t*_ denotes the price of asset *i* at time *t*.

Given a set of *n* assets, the VAR model represents the log returns of these assets as:

$${𝐫}_{t}=𝐜+{𝐀}_{1}{𝐫}_{t-1}+{𝐀}_{2}{𝐫}_{t-2}+\ldots +{𝐀}_{p}{𝐫}_{t-p}+{𝐮}_{t}.$$ (5)

The variable ${𝐫}_{t}$ is the vector of log returns at time *t*,

𝐜 is the vector of intercepts,

${𝐀}_{1},\ldots ,{𝐀}_{p}$ are the coefficient matrices, and ${𝐮}_{t}$ is the vector of error terms, assumed to be white noise with covariance matrix Σ.

The OLS method is commonly used to estimate the parameters of the VAR model. The model also enables the examination of impulse response functions. Forecast error variance decomposition is a method used to analyse the impact of shocks to individual assets and how they contribute to the forecast error variances of all assets [19–21].

### Principal component analysis

PCA is a statistical method commonly used for dimensionality reduction in a variety of domains, including financial mathematics. In the context of portfolio optimisation, PCA can be of great assistance in the process of extracting dominating financial ratios from a wide variety of economic data. These ratios can then be used to design an optimal investment portfolio [24]. The power of PCA in the field of financial mathematics stems from its capacity to transform complex multidimensional data into information easier to manage without compromising essential information. This, in turn, enables better informed investment decisions. It is interesting to note that although PCA has traditionally been linked to the maximisation of variance, greater variance does not always signal more important information for portfolio optimisation [24].

PCA is a prominent statistical methodology that employs an orthogonal transformation to transform a set of correlated observations of multiple variables into a set of principal components, which are linearly uncorrelated values. This procedure is frequently used to decrease the number of dimensions in datasets, improving comprehensibility while minimising the loss of information [25].

Prior to PCA analysis, a dataset is commonly denoted as an *n* × *p* matrix referred to as 𝐗. Each row in this matrix represents a unique replication of the experiment, while each column represents a specific type of feature, such as data collected from a particular sensor [26]. To achieve precise outcomes, the dataset undergoes preprocessing to ensure that the empirical mean of each column is zero, suggesting that the sample mean of each characteristic has been corrected to zero.

The transformation in PCA is determined by a collection of *l p*-dimensional weight vectors or coefficients, denoted as ${𝐰}_{\left(k\right)}=(w_{1},\ldots ,w_{p}{)}_{\left(k\right)}$. The weight vectors are used to transform each row vector ${𝐱}_{\left(i\right)}$ of 𝐗 into a new vector ${𝐭}_{\left(i\right)}=(t_{1},\ldots ,t_{l}{)}_{\left(i\right)}$ of principal component scores, which are calculated as follows:

$$t_{k(i)}={𝐱}_{\left(i\right)}\cdot {𝐰}_{\left(k\right)}\;\text{for}\;i=1,\ldots ,n\;\text{and}\;k=1,\ldots ,l.$$ (6)

The scores $t_{1},\ldots ,t_{l}$ are calculated in a way that maximises the variance they derive from 𝐗, while ensuring that each weight vector 𝐰 is a unit vector.

To maximise variance, the initial weight vector ${𝐰}_{\left(1\right)}$ is obtained by solving the following optimisation problem:

$${𝐰}_{\left(1\right)}=\arg\max_{\|𝐰\|=1}\left\{\sum_{i}(t_{1(i)}{)}^{2}\right\}=\arg\max_{\|𝐰\|=1}\left\{\sum_{i}({𝐱}_{\left(i\right)}\cdot 𝐰{)}^{2}\right\}.$$ (7)

Alternatively, this can be represented in matrix format as:

$${𝐰}_{\left(1\right)}=\arg\max_{\|𝐰\|=1}\left\{\|Xw{\|}^{2}\right\}=\arg\max_{\|𝐰\|=1}\left\{{𝐰}^{𝖳}{𝐗}^{𝖳}Xw\right\}.$$ (8)

The first principal component score for a data vector ${𝐱}_{\left(i\right)}$ is determined by the dot product of ${𝐱}_{\left(i\right)}$ and ${𝐰}_{\left(1\right)}$, resulting in $t_{1}(i)={𝐱}_{\left(i\right)}\cdot {𝐰}_{\left(1\right)}$.

The *k*-th principal component can be obtained by first removing the influence from the previous *k*−1 components. This modified data matrix, denoted as ${\hat{𝐗}}_{k}$, is written as:

$${\hat{𝐗}}_{k}=𝐗-\sum_{s=1}^{k-1}𝐗{𝐰}_{\left(s\right)}{𝐰}_{\left(s\right)}^{⊤}.$$ (9)

Here, ${𝐰}_{\left(s\right)}$ represents the weight vectors based on the first *k*−1 principal components.

The weight vector for the *k*-th principal component, ${𝐰}_{\left(k\right)}$, is then determined by solving the following optimisation problem:

$${𝐰}_{\left(k\right)}=\arg\max_{\|𝐰\|=1}\left\{\|{\hat{𝐗}}_{k}𝐰{\|}^{2}\right\}=\arg\max\left\{\frac{{𝐰}^{⊤}{\hat{𝐗}}_{k}^{⊤}{\hat{𝐗}}_{k}𝐰}{{𝐰}^{⊤}𝐰}\right\}.$$ (10)

Solving this maximisation optimisation problem yields an eigenvector that has a strong relationship with the largest eigenvalue of ${\hat{𝐗}}_{k}^{⊤}{\hat{𝐗}}_{k}$, which helps to determine the direction of maximum variance.

The *k*-th principal component score for a data vector 𝐱(i) can be evaluated as:

$$t_{k}(i)=𝐱(i)\cdot 𝐰(k).$$ (11)

Here, 𝐰(k) represents the *k*-th eigenvector of ${𝐗}^{⊤}𝐗$.

The complete principal component decomposition of 𝐗 can be written as:

𝐓=𝐗𝐖. (12)

In this context, the matrix of weights, denoted as 𝐖, consists of columns that are the eigenvectors of ${𝐗}^{⊤}𝐗$. These columns, which are scaled by the square root of the corresponding eigenvalues, are referred to as loadings.

The covariance matrix, denoted by 𝐐, is proportional to the transpose of the matrix X multiplied by ${𝐗}^{⊤}𝐗$. Specifically, 𝐐 is related to the PCA of the dataset, and the covariance between different principal components can be expressed as follows [26]:

$$Q({PC}_{\left(j\right)},{PC}_{\left(k\right)})=\lambda_{\left(k\right)}{𝐰}_{\left(j\right)}^{⊤}{𝐰}_{\left(k\right)}.$$ (13)

The eigenvalues are represented by the symbol $\lambda_{\left(k\right)}$. The eigenvectors corresponding to various eigenvalues are orthogonal, which ensures there is no covariance between different principal components.

The matrix transformation that diagonalises the empirical sample covariance matrix is often referred to as the whitening or sphering transformation. In formal terms, it is represented as follows:

$${𝐖}^{⊤}𝐐𝐖\propto \Lambda .$$ (14)

Here, Λ represents the diagonal matrix of eigenvalues.

PCA is an effective method for transforming original variables into a new coordinate system. In this new system, the axes are the directions of maximum variance, which are orthogonal to each other and ranked according to the variance they explain. This transformation is essential for applications in data reduction, noise reduction, and exploratory data analysis.

PCA transforms a data vector 𝐱(i) from the original space defined by p variables into a new space of *p* uncorrelated variables. This transformation is defined by the matrix equation:

𝐓=𝐗𝐖. (15)

This equation represents the relationship between the original data matrix (denoted as 𝐗) and the matrix of eigenvectors (denoted as 𝐖) ,where the columns of 𝐖 are the eigenvectors of the covariance matrix of 𝐗.

However, it is not mandatory to preserve all principal components. Preserving only the first *L* principal components, which correspond to the first *L* eigenvectors, leads to a truncated transformation:

$${𝐓}_{L}=𝐗{𝐖}_{L},$$ (16)

where ${𝐓}_{L}$ contains *n* rows but only *L* columns, effectively reducing the dimensionality of the data. The transformation yielded by PCA takes the following form:

$$𝐭={𝐖}_{L}^{⊤}𝐱,\;𝐱\in {\mathbb{R}}^{p},\;𝐭\in {\mathbb{R}}^{L}.$$ (17)

Here, ${𝐖}_{L}$ represents a p×L matrix that forms an orthogonal basis for the *L* features [27].

The purpose of this transformation is to optimise the score matrix ${𝐓}_{L}$ so that it maximises the amount of variance from the original data that is preserved and minimises the total squared reconstruction error. This can be quantified by the following equation:

$$\|𝐓{𝐖}^{⊤}-{𝐓}_{L}{𝐖}_{L}^{⊤}{\|}_{2}^{2}\;\text{or}\;\|𝐗-{𝐗}_{L}{\|}_{2}^{2}.$$ (18)

The difference between the reconstructed data from the full transformation and that from the truncated transformation is represented by this error measure. This difference highlights the effectiveness of PCA in identifying the most important aspects of the data.

By using PCA, key features of the dataset can be effectively preserved in a smaller number of dimensions. This makes analysis and visualisation less complex, while preserving essential information on the variability of the data.

### Continuous wavelet transform

The CWT can be used to analyse non-stationary signals. This is accomplished by splitting the signals into wavelets, which are functions that are both time- and frequency-localised. Unlike Fourier transforms, the CWT provides a representation of time and frequency, making it particularly well suited for signals whose frequency content is subject to changes over time.

The CWT of a function f(t) is represented as [28]:

$$W_{f}(a,b)=\frac{1}{\sqrt{\left|a\right|}}\int_{-\infty }^{\infty }f(t)\psi^{*}\left(\frac{t-b}{a}\right)\;dt.$$ (19)

The function ψ(t) is the mother wavelet, where *a* and *b* represent the scale and translation parameters, respectively, and $\psi^{*}$ signifies the complex conjugate of ψ [28, 29].

To meet the admissibility requirement—having a zero mean and a finite energy—it is crucial for the mother wavelet ψ(t) to act as a band-pass filter. Furthermore, it needs to be zero-integrated [28, 29].

The signal processing community uses CWT for a variety of tasks, including feature extraction, noise reduction, and time-frequency analysis.

### Recurrent neural networks

An RNN is an artificial neural network in which the connections between nodes follow a specific temporal sequence. This allows them to display temporally dynamic behaviour for the given time sequence. RNNs differ from feedforward neural networks in that they may process input sequences using their internal state (memory). This opens up a world of possibilities for application, such as to speech recognition and unsegmented linked handwriting recognition.

The fundamental building blocks of RNNs are nodes, also known as neurons. At each time step, nodes modify their activation and transmit this activation to the subsequent time step [30, 31].

According to [32], this can be expressed mathematically as follows. The input at each step *t* is represented by each element of the input vector $\left\{x_{1},x_{2},\ldots ,x_{T}\right\}$. The RNN uses these equations to find the hidden vector sequence $\left\{h_{1},h_{2},\ldots ,h_{T}\right\}$ and, if necessary, the output vector sequence $\left\{y_{1},y_{2},\ldots ,y_{T}\right\}$ from *t* = 1 to *T*:

$$h_{t}=\sigma (W_{hh}h_{t-1}+W_{xh}x_{t}+b_{h})\;\;\text{and}$$ (20)

$$y_{t}=W_{hy}h_{t}+b_{y}.$$ (21)

Here, the hidden state at time *t* is represented by *h*_*t*_. The activation function is usually a non-linear function like tanh or ReLU, and the weight matrices (parameters) for input-to-hidden, hidden-to-hidden, and hidden-to-output connections are *W*_*hh*_, *W*_*xh*_, and *W*_*hy*_, respectively.

The bias vectors are denoted as *b*_*h*_ and *b*_*y*_, whereas the output at time *t* is represented by *y*_*t*_.

In the case of output gradients that are very small relative to the parameters, the vanishing gradient problem arises, making the network incapable of identifying long-range correlations in the input data. This is one of the main obstacles to training RNNs. According to [30], two common RNN variations that aim to address this problem are LSTM units and GRUs.

LSTM networks also employ gating mechanisms to control the information flow, and they keep a separate cell state in addition to the concealed state. A set of gate types are present in every unit. A forget gate determines which pieces of data to remove from the cell’s state, while an input gate determines which cell state values should be updated.

**Fig 1:**
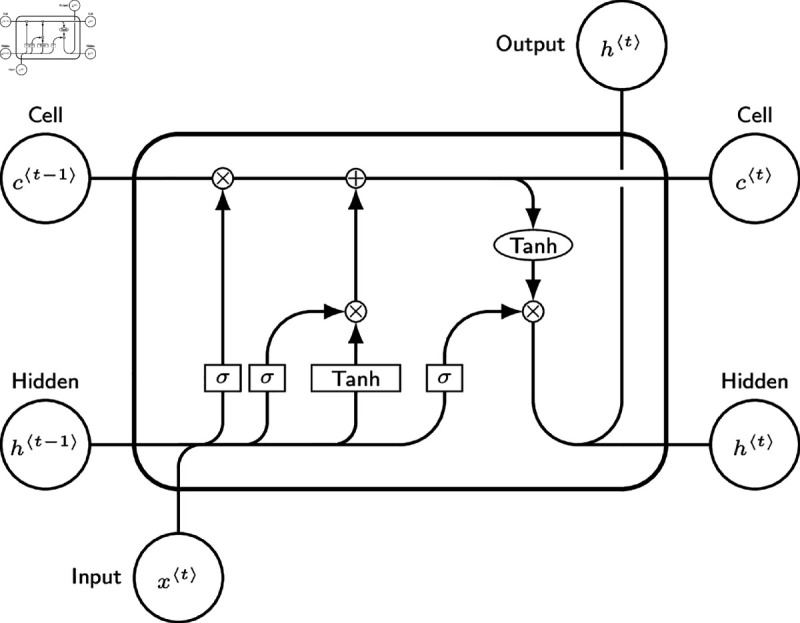
An LSTM model diagram by J. Leon, Beerware. The graph shows the the architecture of LSTM model.

It is the job of the output gate to decide at each step which component of the cell state should be output see (Fig 1).

The mathematical expressions for these gates are as follows:

$$f_{t}=\sigma (W_{f}\cdot [h_{t-1},x_{t}]+b_{f}),$$ (22)

$$i_{t}=\sigma (W_{i}\cdot [h_{t-1},x_{t}]+b_{i}),$$ (23)

$$o_{t}=\sigma (W_{o}\cdot [h_{t-1},x_{t}]+b_{o}),$$ (24)

$${\tilde{c}}_{t}=\tanh(W_{c}\cdot [h_{t-1},x_{t}]+b_{c}),$$ (25)

$$c_{t}=f_{t}\cdot c_{t-1}+i_{t}\cdot {\tilde{c}}_{t}\;\;\text{and}$$ (26)

$$h_{t}=o_{t}\cdot \tanh(c_{t}).$$ (27)

### Convolutional neural networks

**Fig 2:**
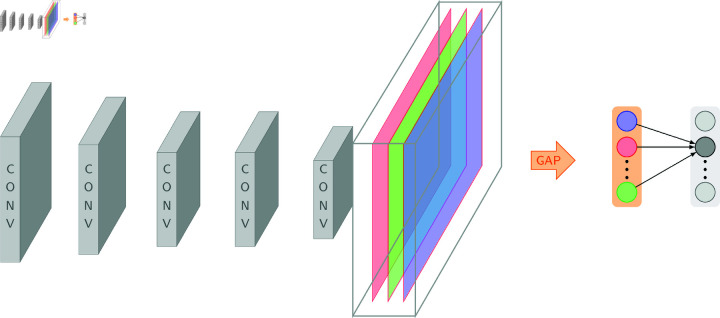
A CNN architecture illustrution [36]. The graph shows the the architecture of CNN model.

Combining concepts from deep learning with signal processing allows for a description of the architecture and functioning of CNNs using wavelet transforms. This is especially important when dealing with financial time series data, such as *r* log returns. When analysing non-stationary financial time series data, in which features such as volatility might fluctuate over time, wavelet transforms offer a highly beneficial time-frequency analysis tool see (Fig 2).

This article uses CNNs in conjunction with the CWT to analyse financial time series data, with a particular emphasis on r(t) log returns. Improved CNN feature extraction for dynamic financial data is achieved using the fine-grained time-frequency localisation features of CWTs.

Financial instruments’ log returns are notoriously difficult to analyse due to their non-linear behaviour and volatility. The CWT is one effective method for breaking down these non-stationary signals into time-frequency space and improving the capacity to detect localised scale-dependent patterns.

A time series r(t) can be transformed into a two-dimensional signal representation in the time and frequency domains using the CWT [33, 34]. The definition of the transform is:

$$R(a,b)=\int_{-\infty }^{\infty }r(t)\frac{1}{\sqrt{a}}\psi \left(\frac{t-b}{a}\right)\;dt,$$ (28)

where *a* and *b* are the scale and translation parameters, respectively, and ψ(t) is the mother wavelet function.

The CNN receives its input from the two-dimensional CWT coefficient matrix, which encodes signal information across both time and frequency.

To extract features from the CWT coefficients, these layers employ various filters via the equation:

$$a_{i}^{\;[l+1]}=\sigma \left(b_{i}^{\;[l]}+\sum_{j}f_{ij}^{\;[l]}\cdot a_{j}^{\;[l]}\right).$$ (29)

Each filter picks up on unique patterns, such as spikes in frequency around certain dates, which may be indicative of major financial occurrences [35]. ${ℛ}_{i}$ represents the region (or set of indices) over which the pooling is performed for the *i*-th unit of layer *l* + 1.

The deep neural network is completed by fully linked layers that combine the acquired characteristics to make predictions or classify patterns.

During training, the goal is to improve a loss function suitable for the specific job at hand, such as mean squared error for regression or cross-entropy for classification. This is achieved by employing methods such as stochastic gradient descent.

Combining CWTs with CNNs provides an effective method for analysing complex and dynamic financial time series. This strategy improves the accuracy of predictions and allows for improved extraction of data by collecting specific local characteristics at different levels of detail [34].

## Fourier–Mellin transform

This section provides an examination of the image-matching methodology (in this context, by image, we mean the log-returns time series lag of the asset’s wavelet transform), showing its limitations and offering the symmetry phase-only FMT (SPOFM) as a potential alternative approach. Two phases are involved in SPOFM: calculating the Fourier–Mellin invariant (FMI) descriptors and matching FMI descriptors to two-dimensional images. Some of the notable advantages of SPOFM are its robustness against noise, accuracy in numerical computations, and effectiveness in data selection. Nevertheless, there are several limitations associated with its use in scenarios involving image rotation and scaling. A proposed alternate approach involves employing a circular harmonic expansion technique, which introduces the additional task of identifying the common centre of rotation. The combination of the FMI and SMPOF methodologies capitalises on their respective strengths and is experiencing growing popularity. This section also examines the role of the FMT in the comparison of image sizes, emphasising its wide peak as a notable limitation in determining the location and identification of object elements.

The primary aim of the image comparison is the recognition of the existence of an image within a background of noise in the form:

$$s(x,y)=r(x^{\prime },y^{\prime })+n(x,y).$$ (30)

The term on the left-hand side is the noisy scene function *s*(*x*, *y*). The first function on the right-hand side, $r(x^{\prime },y^{\prime })$, is the image function, while the remaining function *n*(*x*, *y*) is the zero mean, which remains unaffected by the image signal *r*(*x*, *y*). The following equation can be used to determine the geometric translation measures between the two images:

$$s(x,y)=r(x^{\prime }(x,y,p_{1},\ldots ,p_{n}),y^{\prime }(x,y,p_{1},\ldots ,p_{n}))+n(x,y).$$ (31)

Here, the $x^{\prime }$ and $y^{\prime }$ components, as well as the geometric components $p_{1},p_{2},\ldots ,p_{n}$, represent the computational functions associated with the geometric translation regarding the variable *p*. For illustrative purposes, consider a two-dimensional scenario where the translation offset is denoted as $\left(x_{0},y_{0}\right)$.

R(u,v)=ℱ(r(x,y)),S(u,v)=ℱ(s(x,y)). (32)

Here, ℱ is the Fourier transform function, and:

$$x^{\prime }=x-x_{0},\;y^{\prime }=y-y_{0}.$$ (33)

The transfer function *H*(*u*, *v*) can be considered as the maximum detection of the signal, which at the same time minimises the noise. Mathematically, the equation can be expressed as:

$$H(u,v)=\frac{R^{*}(u,v)}{|N(u,v){|}^{2}}.$$ (34)

The quantity $R^{*}(u,v)$ can be defined as the complex conjugate of the Fourier spectrum *R*(*u*, *v*). Additionally, it can be expressed as the product of the noise power-spectral intensity ${\left|N(u,v)\right|}^{2}$.

If the noise spectrum exhibits a uniform distribution, with intensity denoted as *n*_*w*_, then the transfer function decreases to a level determined by:

$$H(u,v)=\frac{1}{|n_{w}{|}^{2}}R^{*}(u,v).$$ (35)

The result of the filter function is given by the convolution of the functions *s*(*x*, *y*) and ${\text{r}}^{*}(-\text{x},-\text{y})$, which can be expressed as:

$$q_{0}(u,v)=\frac{1}{|n_{w}{|}^{2}}\int \int_{-\infty }^{\infty }s(a,b)r^{*}(a-x,b-y)dadb.$$ (36)

The function achieves its highest value at the point $\left(x_{0},y_{0}\right)$, according to [37]. This point determines the values of the translation parameters as well as the intensity of the noise, denoted as *n*_*w*_ and calculated as the squared absolute value of *N*(*u*, *v*).

This becomes an obstacle in image matching approaches when there is limited shape differentiation but where the images have the same size and energy content [37].

This also presents a difficulty in identifying the maximum value in the presence of noise, a problem that can be resolved by the use of a phase-only matching filter.

The transfer function is defined as follows:

$$H(u,v)=\text{Phase}(R^{*}(u,v))=\exp[j(-\phi_{r}(u,v))].$$ (37)

In this context, it becomes clear that *j*^2^ = −1 due to the fact that the spectral phase preserves only the spatial information of the object, while rejecting the image’s energy content.

The use of the phase-only matched filter yields a more distinguishable picture in comparison to the conventional matched filtering technique, hence increasing the ability to discriminate between objects.

The approach can be enhanced by acquiring the correlation phase of both the image and the noise function [37]. This is achieved by including a non-linear filter that produces output according to the following equation:

$$Q(u,v)=\frac{S(u,v)}{\left|S(u,v)\right|}•\frac{R^{*}(u,v)}{\left|R^{*}(u,v)\right|}=\exp[j(\phi_{s}(u,v)-\phi_{r}(u,v))].$$ (38)

The spectral phases $\phi_{r}(u,v)$ and $\phi_{s}(u,v)$ indicate the image signal *r*(*x*, *y*) and the noise function *s*(*x*, *y*), respectively. When the level of noise is insignificant, the aforementioned equation can be rewritten as:

$$Q(u,v)=\exp[-j2\pi (ux_{0}+vy_{0})].$$ (39)

The inverse Fourier transform of the above function yields a Dirac delta function centred at the coordinates $\left(x_{0},y_{0}\right)$. This function exhibits superior performance in comparison to phase-only matched filtering (POMF).

The technique discussed here offers as an alternative approach to POMF known as symmetry POMF (SPOMF) [37].

The image transformation method consists of three sequential processes: rotation, scaling, and translation. These steps are applied to an image denoted as *r*(*x*, *y*). The technique involves first collecting the phases of the image, followed by performing POMF [37].

This approach can be achieved by the implementation of pre-processing techniques in the spectral phase.

In the given scenario, an object indicated as *s*(*x*, *y*) performs a series of operations resulting in the generation of a comparable image denoted as *r*(*x*, *y*):

$$\begin{array}{c} s(x,y)=r[\sigma (x\cos\alpha +y\sin\alpha )-x_{0}\;\text{and}\\ \sigma (-x\sin\alpha +y\cos\alpha )-y_{0}]. \end{array}$$ (40)

In the provided context, α represents the rotation angle, and σ denotes the uniform scale factor.

The variables *x*_0_ and *y*_0_ represent the offsets of the transformation. The mathematical representation of the Fourier transform is given by:

$$\begin{array}{c} S(u,v)=\exp^{-j\phi_{s}}\sigma^{-2}|R[\sigma^{-1}(u\cos\alpha +v\sin\alpha ),\\ \sigma^{-1}(-u\sin\alpha +v\cos\alpha )]|. \end{array}$$ (41)

The function $\phi_{s}(u,v)$ characterises the spectral phase of the input image, which is influenced by rotation, scaling, and translation. On the other hand, the spectral magnitude remains unaffected by changes in spatial size:

$$\begin{array}{c} |S(u,v)|=\sigma^{-2}|R[\sigma^{-1}(u\cos\alpha +v\sin\alpha ),\\ \sigma^{-1}(-u\sin\alpha +v\cos\alpha )]|. \end{array}$$ (42)

The scaling is constant under spectral operations for u=v=0, as it is proportional to $\sigma^{-1}$. The two primary processes can be identified by employing polar coordinates to specify the magnitudes of *s* and *r*.

$$\begin{array}{c} r_{p}(\theta ,\rho )=|R(\rho \cos\theta ,\rho \sin\theta )|\;\text{and}\\ s_{p}(\theta ,\rho )=|S(\rho \cos\theta ,\rho \sin\theta )|. \end{array}$$ (43)

It is well established that the spectral magnitude exhibits periodic behaviour with respect to the angle θ. Thus, it is possible to estimate the transfer function of a filter by using only half of the magnitude field, as long as the original image is authentic. Therefore,

$$s_{p}(\theta ,\rho )=\sigma^{-2}r_{p}(\theta -\alpha ,\frac{\rho }{\sigma }).$$ (44)

The function $s_{p}(\theta ,p)$ performs angular rotation, where *r*_*p*_ represents the spectral magnitudes. Conversely, the process of scaling involves the reduction of coordinates while simultaneously amplifying the constant factor denoted as $\sigma^{2}$.

The reduction of scaling can be achieved by implementing logarithms of scale in the radial coordinates, as proposed by [37]. These logarithmic transformations are defined as:

$$r_{\text{pl}}(\theta ,\lambda )=r_{p}(\theta ,\rho ).$$ (45)

Here pl stands for polar-logarithmic representation, and

$$s_{\text{pl}}(\theta ,\lambda )=s_{p}(\theta ,\rho )=\sigma^{-2}r_{\text{pl}}(\theta -\alpha ,\lambda -k).$$ (46)

Here, λ=log(p), and κ=log(σ).

In the given equation, which is presented in polar logarithmic form, the operations of scaling and rotation have been simplified to translation. The resulting equation can be expressed as follows:

$$S_{\text{pl}}(v,\varpi )=\sigma^{-2}\exp^{-j2\pi (v\kappa +\varpi \alpha )}R_{\text{pl}}(v,\varpi ).$$ (47)

Additionally, the computations for rotation and scaling are performed independently. Therefore, this numerical approach can be considered dependable. The natural visual system has significant parallels to the log-polar mapping technique.

The many strategies that have been mentioned can be integrated together to obtain an optimal outcome [37]. The comparison of the FMI descriptor of an image may be made with a variety of methods, including cross-correlation (CC), MF, POMF, and SPOMF. SPOMF exhibits a pronounced correlation peak, as demonstrated in the works of [37].

Let *s*(*x*, *y*) and *r*(*x*, *y*) denote the images obtained from the application of the SPOMF algorithm. Then:

$$Q_{0}(v,\varpi )=\frac{R_{\text{pl}}^{*}(v,\varpi )•S_{\text{pl}}(v,\varpi )}{\left|R_{\text{pl}}^{*}(v,\varpi )|•|S_{\text{pl}}(v,\varpi )\right|}.$$ (48)

The fundamental procedure for the SPOMF of the FMI involves the following steps:

**Algorithm 1 The core mechanism of the FMI-SPOMF algorithm**.

1: The procedure begins by applying the Fourier transform to the FMI descriptor of the reference image, which is represented as $R_{\text{pl}}(v,\varpi )$.

2: After that, it proceeds to extract the phase $\exp[-j\phi_{r}(v,\varphi )]$ from $R_{\text{pl}}(v,\varphi )$.

3: Then, the Fourier transform $S_{\text{pl}}(v,w)$ of the FMI descriptor of the observed image *s*(*x*, *y*) is computed.

4: This is followed by the extraction of the phase:


$$\exp[-j\phi_{s}(v,\varpi )]\;\text{of}\;S_{\text{pl}}(v,\varpi ).$$

5: The output of the SPOMF can be determined using the following equation:


$$Q_{0}(v,\varpi )=\exp[-j(\phi (v,\varpi )-\phi_{r}(v,\varpi ))].$$

6: The process for determining the inverse of the Fourier transform is obtained via


$$q_{0}(\theta ,\lambda )={ℱ}^{-1}(Q_{0}(v,\varpi )).$$

7: The final procedure is to locate the point where the function $q_{0}(\theta ,\lambda )$ is at its maximum.

In the initial phase of the procedure, it is assumed that all the images have identical characteristics, with the fundamental aim being to identify the geometric transformation that establishes correlation between these images.

The execution of the core FMI-SPOMF algorithm is performed as follows:

1. The output of the maximum filter is denoted as $q_{0}(\theta ,\lambda )$ and is subsequently identified at the precise coordinates $\theta =\theta_{max}$ and $\lambda =\lambda_{max}$.
2. The value of the rotational angle is denoted as α and is equal to the maximum angle of rotation, represented by $\theta_{max}$.
3. The scaling factor, denoted as σ, is mathematically defined as the exponential function of the maximum eigenvalue $\lambda_{max}$.

The descriptor is generated using half of the spectrum, resulting in an estimated rotation angle ranging from 0 to 180 degrees. The actual angle of rotation depends on a pair of potential values: α or $180^{\circ }$  +  α. The second stage involves the use of the image registration method to determine the translation offset, followed by the determination of the rotation angle. A scaling process is performed on the observed image, resulting in two re-scaled images. These re-scaled images are then rotated by an angle of $\alpha +180^{\circ }$. When executed accurately, the spectrum of the rotated image exhibits a phase discrepancy with *R*(*u*, *v*), which results from the translation of the image. When performed incorrectly, there is a lack of relationship between the spectrum and the coordinates, denoted as *R*(*x*, *y*).This implies that the appropriate angle of rotation can be calculated. This approach can be effectively employed in the identification of pattern recognition problems.

We can now go on to clarify the structure of image registration. Algorithm 2 below outlines the stages involved in image registration using the FMI-SPOMF.

**Algorithm 2 Image registration mechanism.**

1: Initiate the core FMI-SPOMF algorithm.

2: Identify the location coordinates (α,κ) that correspond to the maximum of $q_{0}(\theta ,\lambda )$.

3: Resize the image *s*(*x*, *y*) by a factor of $\sigma^{-1}$ and repeat this process.

4: Generate two resized versions of the image and rotate them by α and $180^{\circ }+\alpha$, respectively.

5: Assess the SPOMF between the reference image *r*(*x*, *y*) and the resized, rotated versions of the observed image.

6: Among the two manipulated images, determine the one with the highest filter maximum.

7: Identify the coordinates of this maximum.

8: Use the geometric features obtained from this transformation process.

Having discussed the importance of every element within the image registration framework, we can go on to outline an application that demonstrates how to take advantage of these geometric characteristics to optimise a portfolio.

To obtain these geometric characteristics, we employ the CWT on *R*_*pca*_ in a process similar to that described in Algorithm 4. However, it is necessary to provide a reference dataset to implement Algorithm 2.

## System design

This section provides a comprehensive explanation of the system’s architecture and capabilities, offering a comprehensive understanding of the interactions across its components. This is of crucial significance to developing an in-depth knowledge of the operational dynamics of the system and the impact of design choices on its performance.

Within the scope of our research, the system being analysed comprises three separate investing strategies: VAR(1)-AutoML, CWT-CNN, and FM-LSTM. These methods reflect the system developed for forecasting financial markets and maintaining investment portfolios.

The first element of our system, known as VAR(1)-AutoML consists of the following steps. First, let $P\in {\mathbb{R}}^{n\times m}$ denote the matrix of stock prices, where *P*_*ij*_ represents the price of the ${\text{i}}^{\text{th}}$ stock at the ${\text{j}}^{\text{th}}$ time point. Then, we find the log returns matrix $R\in {\mathbb{R}}^{n\times (m-1)}$, which can be calculated as $R_{ij}=\ln\left(\frac{P_{i(j+1)}}{P_{ij}}\right)$. Before applying PCA, the data is standardised. Let $R_{std}\in {\mathbb{R}}^{n\times (m-1)}$ be the standardised log returns matrix, where $R_{std_{ij}}=\frac{R_{ij}-\mu_{i}}{\sigma_{i}}$, and $\mu_{i}$ and $\sigma_{i}$ are the mean and standard deviation of the ${\text{i}}^{\text{th}}$ stock returns, respectively.

After standardisation, PCA can be applied to obtain the matrix $R_{pca}=\text{PCA}(R_{std})$, where $R_{pca}\in {\mathbb{R}}^{n\times k}$, and k≤m − 1 is the number of principal components chosen. The method of using retained principal components is called Kaiser’s rule. According to this rule, it is recommended to retain only those components with eigenvalues exceeding 1, since they provide a greater quantity of information compared to single variables.

To effectively capture the temporal dynamics of stock prices and predict future returns, we use a rolling window method for VAR modelling. The VAR(1) model, a multivariate extension of the standard autoregressive model, is chosen for its capacity to represent the linear relationship present across numerous time series.

The method we developed is designed to calculate a series of VAR(1) models. Each model is built using a rolling window approach, where the window includes the most recent *L* = 22 trading days. The decision to use *L* coincides with the normal trading procedure, wherein each model is updated using a dataset that covers approximately 30 days of current data. The use of a rolling window method allows our system to respond effectively to fluctuations in market dynamics, as the estimated model parameters possess the ability to fluctuate over time.

The VAR(1) operates as follows:

1. The system is initialised by specifying an empty VAR(1) model structure *V* with *p* variables, where *p* is the number of stocks in the dataset.
2. The following steps are implemented for each trade day *i* (from the day after the initial *L* days to the final day in the dataset):
  - The VAR(1) model *P*_*i*_ is estimated using the logarithmic returns of the *p* stocks, spanning from day *i* to *i* + *L*−1. The above method produces a set of VAR(1) coefficients of the model, which successfully describe the linear relationships between the stocks, using the *L* most recent days of data.
  - A vector *E*_*i*_ is initialised to include the predicted returns for each day *i*.
  - For every day *j* within the range of *i*−*L* + 1 (or day 1, if *i*−*L* + 1 is less than 1) to *i*, a one-step-ahead forecast is calculated using the VAR(1) model *P*_*i*_ and the log returns from day *i* to *i* + *L*−1. The forecasts are collected and stored in the variable *E*_*i*_.
  - Eventually, the predictions collected in *E*_*i*_ are averaged across the number of days used in the estimation, which is the minimum between *i* and *L*. This process results in an average forecast of returns for day *i*, specifically, for a one-step-ahead prediction.

Once this procedure is completed, a time series of expected returns *E* is obtained. Each *E*_*i*_ is calculated from a VAR(1) model, which is estimated using the most recent *L* days of data. The suggested approach combines VAR modelling, with its advantages for capturing linear connection among shares, with a rolling window method to efficiently react to changing market dynamics.

The division of data into training and testing sets is a critical stage in the application of deep learning or machine learning techniques. In this process, the data is partitioned, with 80% allocated for training and the remaining 20% reserved for testing. The purpose of the training dataset is to aid in the training of the system, while the testing dataset is used to assess and evaluate the performance of the model. Algorithm 3 below illustrates the model’s procedure:

**Algorithm 3 VAR(1)-AutoML investment strategy.**

1: **procedure** StockPricePrediction (*P*, *n*, *m*)

2:   Initialise $R\in {\mathbb{R}}^{n\times (m-1)}$

3:   **for** *i* = 1 to *n* **do**

4:    **for** *j* = 1 to *m*–1 **do**

5:     $R_{ij}=\ln\left(\frac{P_{i(j+1)}}{P_{ij}}\right)$

6:     $R_{std_{ij}}=\frac{R_{ij}-\mu_{i}}{\sigma_{i}}$

7:    **end for**

8:   **end for**

9:   $R_{pca}=\text{PCA}(R_{std})$ ⊳ Apply PCA using Kaiser’s Rule

10:   Split *R*_*pca*_ into $R_{pca_{tr}}$ and $R_{pca_{te}}$

11:   $model=\text{fitrauto}(R_{pca_{tr}})$ ⊳ Train the model

12:   Test the model on $R_{pca_{te}}$

13:   Determine important feature order in $R_{pca_{tr}}$ and $R_{pca_{te}}$, relying on the *model* outcomes by masking *R*_*pca*_.

14:   Calculate cumulative log returns as *CL*_*tr*_ and *CL*_*te*_ for $R_{pca_{tr}}$ and $R_{pca_{te}}$, respectively

15:   **return** *CL*_*tr*_, *CL*_*te*_

16: **end procedure**

Important note: fitrauto(…) *is a model that trains with a tool that uses automated procedures to select between Bayesian optimisation and the Asynchronous Successive Halving Algorithm. These algorithms are applied to a range of regression model types with varying hyperparameter values. The output of fitrauto is the model anticipated to yield the most accurate predictions for new data.*

The second suggested approach – CWT-CNN – involves combining a wavelet transform with a CNN. The wavelet transform is used as a first step to divide the investment data into distinct frequency components. Subsequently, a CNN is employed to identify the important features of the data for portfolio optimisation. This model includes an important phase that involves the modification and extraction of features from a dataset with several dimensions. To achieve this, a CWT is performed on specific components of the data collection.

The technique begins by creating new transformed data points, denoted as *Q*, whose dimensions are determined by the span of the dataset dates (which is decreased by *L* elements from the end) and the parameter *p*. The matrix *Q* is arranged to enable the storage of the results of the repeated wavelet transformations.

After the creation of *Q*, the algorithm proceeds to participate in a two-level iteration loop. The outer loop sequentially traverses each element in the dataset *P*, which is a vector that stores the indices of the dataset. For every iteration *i* in the set *P*, a nested loop is initiated, which operates *p* times. The algorithm conducts a CWT on a section of the *j*-th column of the data matrix *R*_*pca*_, covering the *i*-th row to row *i* + *L*−1, within the nested loops.

The proposed approach involves the application of a CWT to different sections of the multi-dimensional dataset. The results of the transformation outputs are important for capturing vital information from the data. The feature extraction technique plays a crucial role in the data analysis pipeline of the system as a whole.

**Algorithm 4 CWT-CNN investment strategy.**

1: **procedure** StockPricePrediction (*P*, *n*, *m*)

2:   Initialise $R\in {\mathbb{R}}^{n\times (m-1)}$

3:   **for** *i* = 1 to *n* **do**

4:    **for** *j* = 1 to *m*–1 **do**

5:     $R_{ij}=\ln\left(\frac{P_{i(j+1)}}{P_{ij}}\right)$

6:     $R_{std_{ij}}=\frac{R_{ij}-\mu_{i}}{\sigma_{i}}$

7:    **end for**

8:   **end for**

9:   $R_{pca}=\text{PCA}(R_{std})$ ⊳ Apply PCA using Kaiser’s rule.

10:   Apply CWT on *R*_*pca*_.

11:   Split *R*_*pca*_ into $R_{pca_{tr}}$ and $R_{pca_{te}}$.

12:   $model=\text{CNN}(R_{pca_{tr}})$ ⊳ Train the model.

13:   Test the model on $R_{pca_{te}}$

14:   Determine important feature order in $R_{pca_{tr}}$ and $R_{pca_{te}}$, relying on the *model* outcomes by masking *R*_*pca*_.

15:   Calculate cumulative log returns as *CL*_*tr*_ and *CL*_*te*_ for $R_{pca_{tr}}$ and $R_{pca_{te}}$, respectively.

16:   **return** *CL*_*tr*_, *CL*_*te*_.

17: **end procedure**

The third component, the FM-LSTM, implements the FMT in conjunction with an LSTM network. The FMT is employed to transform the time-series investment data into a representation that is invariant to changes in scale and rotation. Following this, an LSTM network is used to find the important features to control and optimise the portfolio.

Here is the outline of the FM-LSTM model:

**Algorithm 5 FMT-LSTM investment strategy.**

1: **procedure** StockPricePrediction (*P*, *n*, *m*)

2:   Initialise $R\in {\mathbb{R}}^{n\times (m-1)}$

3:   **for** *i* = 1 to *n* **do**

4:    **for** *j* = 1 to *m*–1 **do**

5:     $R_{ij}=\ln\left(\frac{P_{i(j+1)}}{P_{ij}}\right)$

6:     $R_{std_{ij}}=\frac{R_{ij}-\mu_{i}}{\sigma_{i}}$

7:    **end for**

8:   **end for**

9:   $R_{pca}=\text{PCA}(R_{std})$ ⊳ Apply PCA using Kaiser’s rule.

10:   Apply CWT on *R*_*pca*_.

11:   Split *R*_*pca*_ into $R_{pca_{tr}}$, $R_{pca_{re}}$ and $R_{pca_{te}}$.

12:   Obtain geometric features from using Algorithm 2 on $R_{pca_{tr}}$ and $R_{pca_{te}}$, relying on $R_{pca_{re}}$.

13:   Assign *FM*_*tr*_ and *FM*_*te*_ as the matrix geometric features from training and testing data, respectively.

14:   $model=\text{LSTM}(FM_{pca_{tr}})$ ⊳ Train the model.

15:   Test the model on $FM_{pca_{te}}$

16:   Determine important feature order in $FM_{pca_{tr}}$ and $FM_{pca_{te}}$, relying on the *model* outcomes by masking *R*_*pca*_.

17:   Calculate cumulative log returns as *CL*_*tr*_ and *CL*_*te*_ for $R_{pca_{tr}}$ and $R_{pca_{te}}$, respectively.

18:   **return** *CL*_*tr*_, *CL*_*te*_.

19: **end procedure**

Here, $R_{pca_{re}}$ is the log return of the PCA reference dataset.

The computational procedures, including data preprocessing, model implementation, and statistical analysis, were performed using custom MATLAB scripts. These scripts are included in the Supporting Information section to ensure reproducibility.

## Results

The portfolio consisted of 1,421 stocks, taken from US stock market between 1 April 2013 and 1 April 2023. The data was obtained from a website reliable for its financial reports and details using the MATLAB connection function whic is available in https://www.mathworks.com/help/datafeed/moneynet.html. The principal purpose of this initial research effort is to experiment with feature-based optimisation approaches. Therefore, the selection was random to ensure a diverse and unbiased sample. This randomness guarantees the assessment of the models avoids the influence of bias from specific sectors or companies.

Additionally, the random precursor allowed for testing of the robustness and generalisation capability of the optimisation models across a broad range of stocks.

We conducted a statistical analysis of the daily prices of the stocks in the portfolio. The aim of the analysis was to identify important features, including the stocks’ central tendency, dispersion, and correlation, to capture important factors for investment strategies and portfolio management.

The investigated statistical measures included the mean, median, standard deviation, skewness, and kurtosis (summary statistic). As mentioned, these describe the central tendency and dispersion of returns. Correlation coefficients were computed to examine the relationships between different stocks.

**Fig 3:**
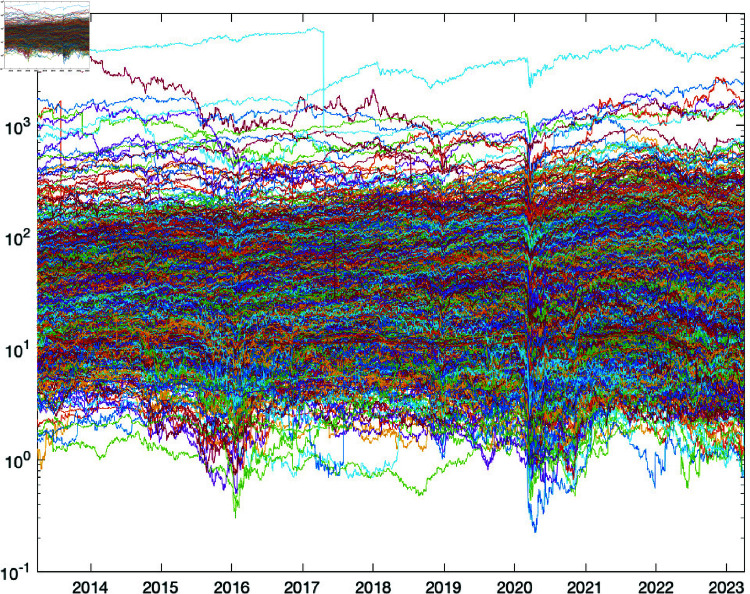
Time series plot of stock prices. The graph shows the performance of the portfolio optimization method using historical stock data.

To obtain an in-depth view of the data, visualisations, including time-series plots, histograms, box plots, and correlation heatmaps were created to provide a graphical representation of these statistics. We began with a visualisation of a time-series plot of the stock prices as presented in (Fig 3) below.

The daily stock prices for the firms under study are summarised in the Table 1 below, along with descriptive information. Mean and median price, standard deviation, skewness, and kurtosis are displayed in separate rows.

**Table 1:** Descriptive statistics on stock prices.

Measure	Min	Max	Mean	Median	StdDev
Mean Prices	2.108	2870.409	60.031	33.238	133.469
Median Prices	1.171	2704.705	54.893	31.430	108.048
Std Dev	0.281	2300.347	25.232	9.736	88.765
Skewness	-3.686	4.866	0.302	0.295	0.653
Kurtosis	1.186	26.017	2.845	2.625	1.232

The average rate of price changes can be calculated using the mean. The median price represents the midpoint of the price distribution and is less volatile than the mean due to extreme values. We found that the average mean and median prices were between 2.108 and 2,870.409 and 1.171 and 2,704.705, respectively.

Using the standard deviation, the volatility of the returns can be assessed. Our research shows that the daily prices of these stocks can vary widely, based on the standard deviation of 0.281 to 2,300.347.

The skewness index quantifies the disproportion in a price distribution. In the case of positive skewness, the tail of the prices is far to the right, whereas in the case of negative skewness, it is far to the left. Our results exhibit skewness in the range of –3.686 to 4.866.

The tailedness of a price distribution can be quantified by its kurtosis. A greater kurtosis suggests a distribution with fatter tails, which may portend more dramatic price swings. Our price kurtosis fell between 1.186 and 26.017 degrees.

These numbers are useful since they shed light on the performance of these equities. There appears to be a wide range of stock performance, as indicated by the wide range of mean and median returns. There also appears to be a wide range of stock risk, as indicated by the large difference in standard deviation. Differences in the distribution of stock returns are further highlighted by the skewness and kurtosis, which exhibit substantial diversity across these equities.

We performed hierarchical clustering based on the correlation matrix of the stock prices calculation. Then, we derived measures from these samples. To obtain these measures through clustering, we applied the so-called linkage method. The figure was truncated to show only a few merges, as the 1,412 samples would be difficult to present using a heatmap.

To implement this approach, we used the following steps based on the daily stocks prices:

1. A correlation matrix among stock prices was computed.
2. A transformation was applied from the correlation matrix to the dissimilarity matrix.
3. The average linkage approach was used to apply hierarchical clustering to the dissimilarity matrix.
4. The resultant dendrogram was truncated so only the most recent *N* mergers are shown.

**Fig 4:**
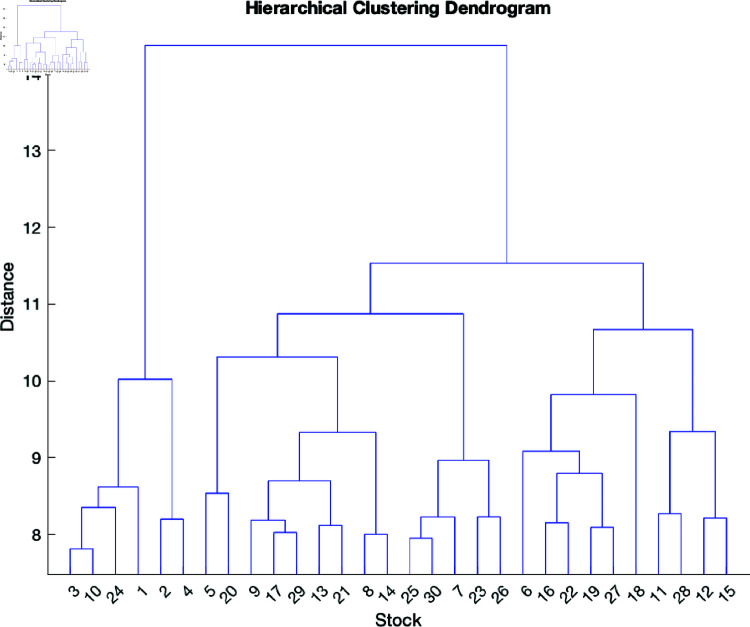
Dendrogram of the stock prices.

The stock returns are displayed in a dendrogram in (Fig 4), which shows their hierarchical grouping. The dendrogram is a tree-like diagram in which each node represents a stock, and the distance between nodes represents the degree of dissimilarity between groups of stocks. To simplify interpretation of the larger clusters, we trimmed the dendrogram to display only the most recent *N* mergers.

To group samples sets, we used an approach that relied on the average correlation with all other samples. The aim of using this approach was to capture the correlation structure in our large datasets comprised of stock prices. The steps for applying this method were as follows:

1. We began by computing the correlation matrix of the dataset using the Pearson correlation coefficient, which measures the linear relationship between two variables. The correlation coefficient ranges from –1 to 1, where –1 indicates a strong negative linear relationship, 1 a strong positive linear relationship, and 0 no linear relationship.
2. We then computed the average correlation for each sample by evaluating the mean of each row in the correlation matrix.
3. We divided the samples into three groups – low, medium, and high – depending on their average correlations. The thresholds for these groups were set randomly and could be adjusted as needed.

**Fig 5:**
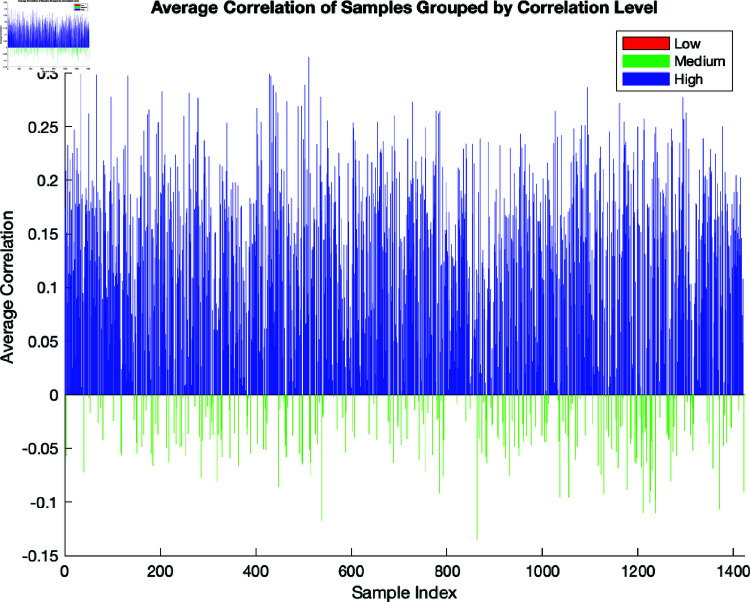
Here, –1, 0, and 1 are the thresholds used to group the correlations.

**Fig 6:**
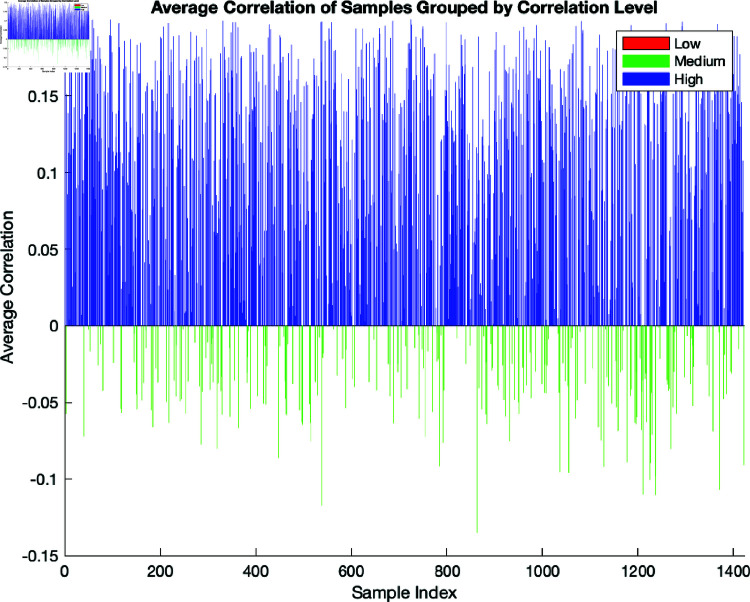
Here, –0.8, 0, and 0.2 are the thresholds used to group the correlations.

Figs 5 and 6 present a bar chart showing the results of the average correlation of samples grouped by correlation level. The x-axis is the sample index, and the y-axis represents the average correlation values. The three groups are designated as follows: The low correlation group is coloured red, the medium group is green, and the high group is blue. The motivation for including this step was to obtain a clear visual representation of the groupings and allow for clear identification of patterns in the data. The fact that the results of averaging and grouping the correlation samples are sensitive to the specific thresholds used for the groupings should also be taken into account.

**Fig 7:**
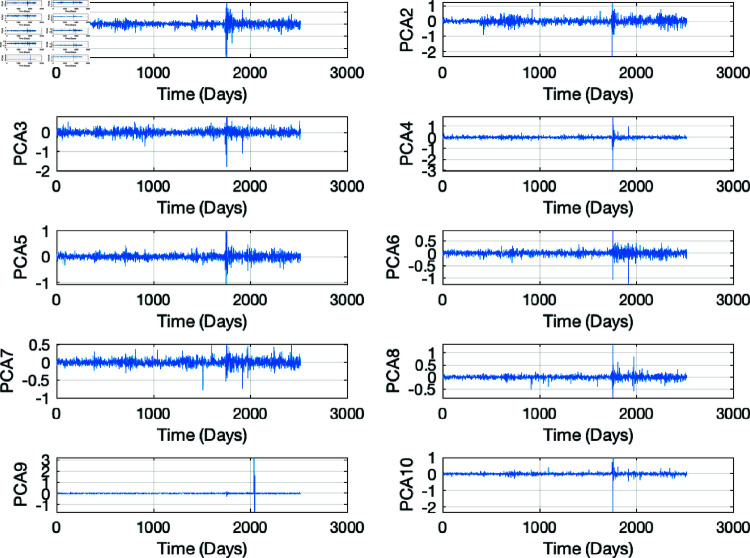
First 10 principal components of the stock log returns.

We also calculated the log returns of the stocks prices and applied PCA to the stocks’ log returns. The Kaiser rule was employed to select the optimal principal components of the data, and the first 10 were kept as a representative of the stock returns of the portfolio. The aim of this strategy was to capture the most variance in the data using the fewest number of principal components. PCA removes outliers and unusual patterns while ensuring noise reduction, as outliers and noise have strong negative effects on identifying the best features for optimising the portfolio. Below is a plot of the first 10 principal components of the data see (Fig 7).

**Fig 8:**
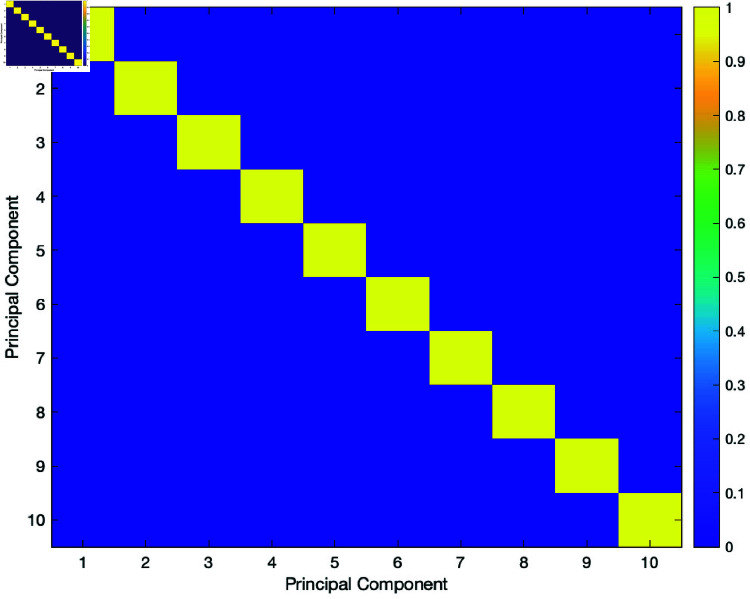
Correlation heatmap of the principal components.

We plotted a heatmap of the correlation matrix for the 10 principal components obtained from the stock log returns derived from the data see (Fig 8). On the heatmap, the diagonal values are equal to 1. This means that each PCA correlated perfectly with itself. The numbers not on the diagonal, which show the relationships between major components, are near zero. This illustrates that these principal components are orthogonal and support the use of PCA. The cause of these small off-diagonal numbers is calculation error or background noise.

**Fig 9:**
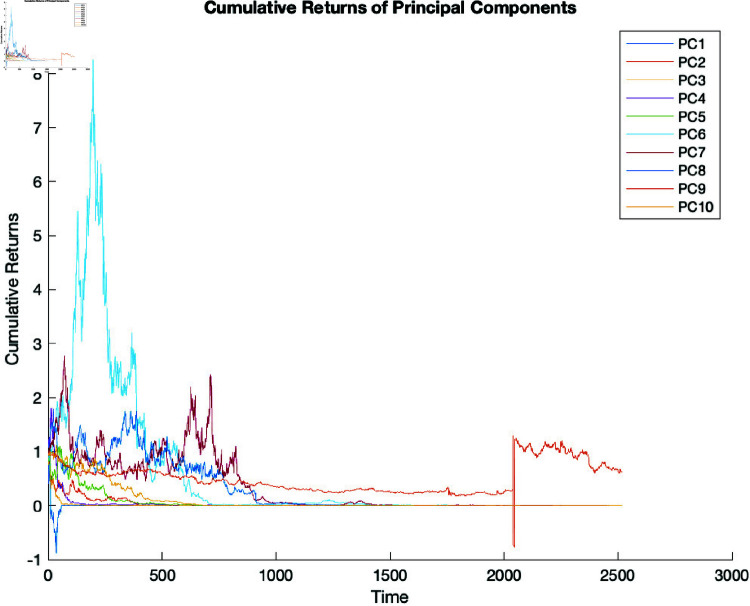
Cumulative returns of the principal components of the log returns.

We also plotted the cumulative log returns of the principal components over time, considering PCA1 to PCA10. (Fig 9) shows that the initial investment was initialised at zero for all cumulative log returns. There are increasing and decreasing log returns over time from 0 to 3,000 days (plotted on the x-axis), which is indicative of the fluctuating nature of returns over time.

The cumulative log returns plotted along the y-axis fall between –1 and 9. Note that a value above zero indicates a positive return, while one below zero indicates a negative return or a loss. These results demonstrate the inherent volatility of the market, with notable fluctuations in cumulative returns for all principal components see (Fig 9).

**Fig 10:**
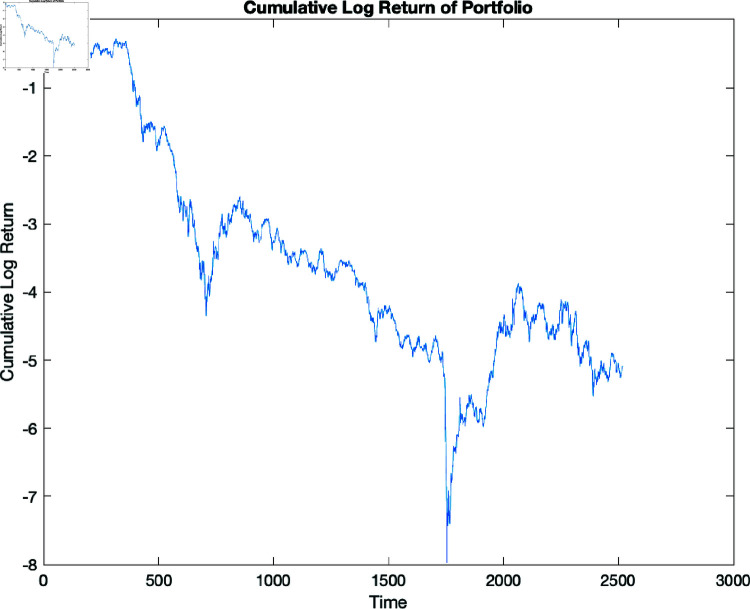
Cumulative returns of a portfolio containing the principal components of the log returns.

We also plotted the cumulative log returns of these 10 principal components, as presented in Fig 10 below:

Fig 10 shows that the portfolio’s cumulative log return decreases over time, with brief intervals of stability marked by less sharp decreases. Portfolio assets are dynamic and variable, which explains these changes. The steeper downwards slopes suggest larger negative returns, whereas the more flat areas indicate relative stability with no substantial positive log returns.

**Fig 11:**
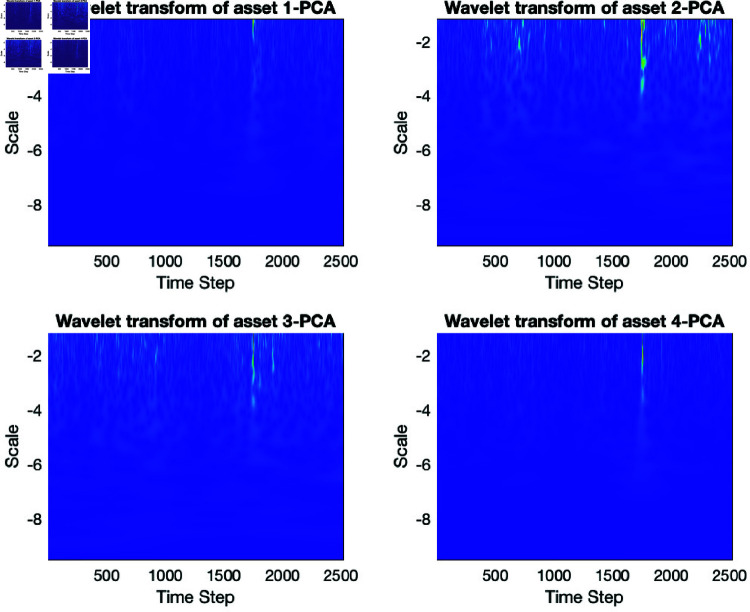
Wavelet transform of some of the principal components of log returns.

A wavelet transform divides a signal into frequency components and analyses each component with a scale-matched resolution. Time-series analysis uses wavelet transform to investigate non-stationary signals, such as financial data see (Fig 11).

To evaluate our model, VAR(1)-AutoML, CWT-CNN, and FM-LSTM were used to analyse the data. We compared the results of these investment approaches by creating profitability plots using cumulative log returns.

**Fig 12:**
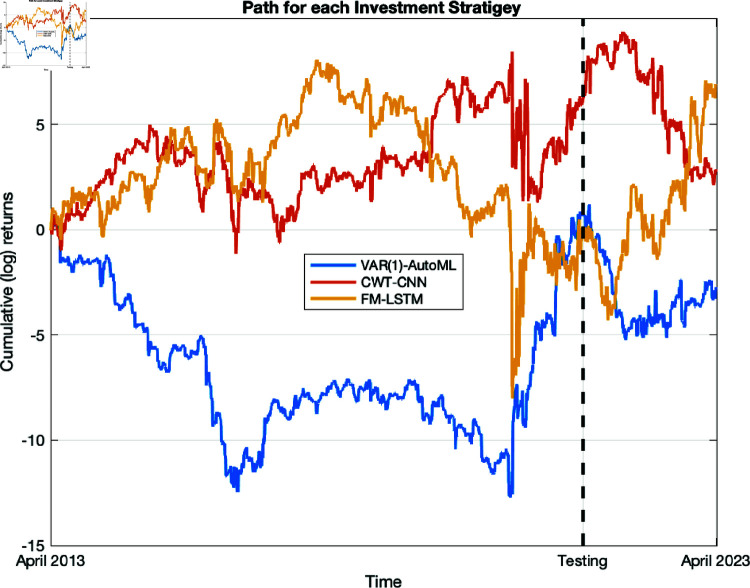
Performance evaluation: the profitability of VAR(1)-AutoML, CWT-CNN, and FM-LSTM.

Fig 12 visualises the paths of the three investment strategies for the 10-year period from April 2013 to April 2023. Cumulative log return was employed for comparative analysis, serving as an indicator of the total investment yield and taking into account the compounding factor.

The *x*-axis of the graph represents the 10-year time span from April 2013 to April 2023. The y-axis represents the cumulative log returns, ranging from –15 to 10. This range signifies the potential for substantial profits, as indicated by positive logarithmic returns, as well as losses, as indicated by the negative logarithmic returns, over a 10-year periods.

The trajectory of each approach on the chart represents its overall performance. The overall patterns indicate varying degrees of effectiveness. The FM-LSTM investment strategy has an upward trajectory on the graph, demonstrating its tendency to generate positive outcomes. In contrast, the VAR(1)-AutoML strategy exhibits periods of decline.

Mean absolute error (MAE) is a statistical metric that quantifies the differences between two observations that represent the same phenomenon. In this context, examples of observations (*Y* versus *X*) include expected and observed values, subsequent time and starting time, or a measuring technique and an alternate measurement approach [38]. We computed the MAE by adding the absolute errors, expressed as the Manhattan distance, and then dividing the sum by the sample size:

$$\mathrm{MAE}=\frac{\sum_{i=1}^{n}\left|y_{i}-x_{i}\right|}{n}=\frac{\sum_{i=1}^{n}\left|e_{i}\right|}{n}$$ (49)

Therefore, the MAE is the geometric mean of the absolute inaccuracies $\left|e_{i}|=|y_{i}-x_{i}\right|$, where *y*_*i*_ is the forecast, and *x*_*i*_ is the actual value. Relative frequencies can be used as weight factors in other formulations. To calculate the MAE, the scale must match that of the observed data. As the MAE is dependent on the scale being used, it cannot be used to compare predicted values measured on different scales [39]. In time series analysis, the MAE is frequently used to measure forecast error and is often confused for the more popular mean absolute deviation. The same misunderstanding is also present more broadly [40].

In statistics, the mean squared error (MSE) or mean squared deviation of an estimator quantifies the average of the squares of the errors, that is, the average squared difference between the estimated values and the actual value [41]. MSE is a risk function that represents the expected value of the squared error loss. The near-constant positivity of MSE, or its tendency not to drop to zero, arises from randomness or the estimator’s inability to incorporate information that could yield a more precise estimate [42, 43]. In machine learning, especially empirical risk minimisation, MSE can be used to represent the empirical risk, which is the average loss on a given dataset [44]. It is a close approximation of the real MSE, which is the average loss on the actual population distribution.

Given a vector of *n* predictions derived from a sample of *n* data points across all variables, where *y*_*i*_ represents the vector of observed values for the predicted variable, and $\hat{y_{i}}$ denotes the predicted values (e.g. obtained from a least-squares fit), the within-sample MSE of the predictor is calculated as [44]:

$$\mathrm{MSE}=\frac{1}{n}\sum_{i=1}^{n}{\left(y_{i}-{\hat{y}}_{i}\right)}^{2}$$ (50)

This study assessed the efficacy of three different investment methods – VAR(1)-AutoML, CWT-CNN, and FMT-LSTM – using MAE and MSE measures across several principal components. The findings shown in Table 2 demonstrate that CWT-CNN consistently achieved the lowest error rates across the majority of components, indicating enhanced prediction accuracy relative to the alternative approaches. Significantly, for principal component 9 (PC9), CWT-CNN exhibited remarkable accuracy, with an MAE of 0.014 and an MSE of 0.001. Conversely, FMT-LSTM demonstrated elevated error rates, especially for PC6 and PC8, indicating possible paths for model enhancement. These findings underscore the efficacy of CWT-CNN for time series prediction tasks in this setting.

**Table 2:** MAE and MSE for the training set across the three methods.

Time Series	VAR(1)-AutoML		CWT-CNN		FMT-LSTM	
	MAE	MSE	MAE	MSE	MAE	MSE
PC1	0.316	0.332	0.336	0.532	0.708	0.930
PC2	0.152	0.073	0.141	0.121	0.168	0.128
PC3	0.138	0.049	0.131	0.065	0.321	0.174
PC4	0.099	0.033	0.092	0.037	0.523	0.336
PC5	0.106	0.030	0.085	0.039	0.122	0.048
PC6	0.102	0.021	0.063	0.016	0.823	0.760
PC7	0.091	0.019	0.073	0.016	0.396	0.204
PC8	0.092	0.020	0.066	0.020	0.838	0.745
PC9	0.066	0.008	0.014	0.001	0.316	0.139
PC10	0.077	0.021	0.057	0.023	Reference	Reference

The findings shown in the testing dataset presented in Table 3 demonstrate that CWT-CNN consistently outperformed the other approaches, yielding reduced error rates for the majority of PCs. CWT-CNN attained the minimum MAE and MSE for PC1 to PC8, indicating its exceptional prediction proficiency. The FMT-LSTM model, although competitive in certain situations (e.g. PC2), typically exhibited elevated error rates, especially for PC6 and PC8. In particular, PC9 was a challenge for all models, exhibiting markedly elevated MSE values. The findings show that the CWT-CNN methodology was highly effective in identifying the fundamental patterns within this dataset, indicating it could improve accuracy in forecasting for analogue time series applications.

**Table 3:** MAE and MSE for the testing dataset across the three methods.

Time Series	VAR(1)-AutoML		CWT-CNN		FMT-LSTM	
	MAE	MSE	MAE	MSE	MAE	MSE
PC1	0.491	2.525	0.464	2.480	0.794	2.833
PC2	0.240	0.139	0.180	0.104	0.195	0.108
PC3	0.174	0.571	0.147	0.565	0.350	0.697
PC4	0.096	0.112	0.090	0.112	0.532	0.431
PC5	0.152	0.040	0.104	0.021	0.139	0.033
PC6	0.132	0.060	0.093	0.049	0.844	0.815
PC7	0.139	0.095	0.101	0.084	0.409	0.262
PC8	0.131	0.179	0.095	0.174	0.873	0.904
PC9	0.536	10.098	0.166	6.371	0.451	6.626
PC10	0.095	0.086	0.068	0.079	Reference	Reference

Fig 12 presents a comparison of the VAR(1)-AutoML, FM-LSTM, and CWT-CNN models in terms of their efficacy in optimising portfolio returns. Under particular circumstances, the FM-LSTM model exhibited exceptional returns, indicating its successful representation of certain market dynamics. Nevertheless, it is important to emphasise that this improved performance does not indicate an innate capacity to effectively forecast future market trends.

The performance outcomes may have been affected by the random decision to use 20% of the data for testing and 80% for training. The variability introduced by this sampling technique could compromise the model results. Although FM-LSTM produced better cumulative returns, its success better indicates flexibility to historical trends rather than forecasting ability.

By contrast, although it did not produce the same degree of returns, the CWT-CNN model provided an insightful analysis of temporal patterns and volatility. We also respect the complexity of financial markets and the restrictions of modelling techniques in dynamics prediction.

Further study is required to investigate the effects of several data splitting cases and improve model resilience. This will assist in understanding the actual predictive power of these models.

## Conclusions and future work

This paper investigated three computational portfolio optimisation strategies employing a PCA of stock log returns. The approaches included VAR(1)-AutoML, CWT-CNN, and FM-LSTM. Each strategy obtained crucial stock return data to enhance the performance of financial portfolio prediction models.

The VAR(1)-AutoML technique made use of VAR(1)’s statistical power for time series forecasting and AutoML’s scalability for model selection and hyperparameter tweaking. This combination accurately captured linear connections and changes in the data over time, providing a solid platform for more complex models.

In contrast, the CWT-CNN approach used the CWT to extract features and decompose financial data series into time-frequency components. Combining these multi-resolution analytical capabilities with the pattern recognition strength of a CNN facilitated the discovery of complex non-linear patterns in data that standard approaches ignore.

After conducting a thorough investigation in the frequency domain using the FMT, the FM-LSTM approach used LSTM networks to model the sequences. This technique helped capture long-term dependencies and cyclic patterns in the data, which are essential for identifying trends and producing more accurate stock performance projections.

While each model has its own benefits, the FM-LSTM model demonstrated promising cumulative log return values, a critical performance indicator in this study. However, the random selection of 20% testing and 80% training data may have influenced the results. For this reason, the results indicate the model’s ability to adapt to these data in the portfolio rather than its prediction capability. Even given this limitation, our model can be considered a potentially beneficial tool for portfolio managers to investigate the the uncertain events in volatile financial markets.

The use of PCA as a preprocessing step reduced dimensionality and highlighted the most important characteristics, improving model performance and portfolio optimisation.

Our work demonstrates how improved data processing and machine learning models can affect financial market forecasts. The efficiency of the FM-LSTM model indicates its potential application in examining and predicting long-term financial trends. A future study could combine these approaches or use additional data sources to improve the stability and adaptability of portfolio optimisation systems. While our model exhibits some predictive power, it is important to consider certain constraints. First of all, the FMT can show sensitivity to noise, limiting the model’s performance on unclean data. When the FMT and LSTM are combined, the data dimensionality may also be reduced to a small number of features, making the prediction process difficult. Particularly in the case of sparse data, the complexity of the integrated model could lead to overfitting. A lack of data points in high-dimensional domains prevents algorithms from identifying significant trends without overfitting. This can significantly impact a model’s generalisability and performance. The issue of dimensionality represents a major obstacle in this study, since it lowers the capacity of predictive models, including LSTM networks, to appropriately forecast financial events. Solving this problem requires increasing the model’s dependability and effectiveness. Feature selection approaches or other dimensionality reduction strategies are essential for this purpose.

To make FM-LSTM models function effectively, it is important to find reference data points that can be used to obtain geometric features from both the training and testing datasets. The number of reference points directly influences the richness of the features obtained, but increasing it may result in higher processing costs and complexity. This requirement imposes a significant limitation, since an excessive number of features may cause overfitting, while too few may result in underfitting and suboptimal model performance. Achieving a balance in the quantity of reference points is critical for improving model precision and efficacy, and careful analysis is required in the design of FM-LSTM applications.

Although our methodology has yielded encouraging results, numerous questions remain unanswered. To enhance the model’s generalisability, it would be beneficial to use a more diverse inter-class asset, such as cryptocurrencies. Additionally, attempting other designs or including different methods, such as mean-variance optimisation, could improve the FM-LSTM model. Combining the GARCH and DCC-GARCH models with FM-LSTM could also help obtain a deep understanding of volatility dynamics. Applying the model to real-time data would further allow for its performance to be tested in dynamic circumstances. Finally, to improve decision-making, it may be useful to check whether the model’s predictions are simple to understand. The proposed models could also be applied in other fields, such as for classification problems in medical diagnosis and epidemics.

## Supporting information

S1 Code main.m This script executes the primary analysis, running the three models and applying profitability, error, training, and testing analyses.
(PDF)

S2 Code stack.m Helper function to stack data into a 2D vector using feature extraction for CNN and LSTM.
(PDF)

S3 Code FM.m Applies the Fourier-Millen transform to extract geometric features.
(PDF)

S4 Code hipass_filter.m High-pass filter function used within FM.m.
(PDF)

S5 Code error_analysis.m Computes MAE and RMSE and plots them as time series.
(PDF)

S6 Code calculateMetrics_csv_1.m Applies MAE and RMSE on VAR(1)-AutoML and CWT-CNN, storing results as CSV files.
(PDF)

S7 Code calculateMetrics_csv_OO.m Applies MAE and RMSE on FM-LSTM.
(PDF)

S8 Code transform_Image.m Transforms 2D images to obtain geometric features.
(PDF)

S9 Code statistical_analysis.m Implements statistical analysis.
(PDF)

S1 File Matlabcode. (PDF)

S2 File Variable table. (PDF)
