# Supplementary material for: Investigating the dynamics and uncertainties in portfolio optimization using the Fourier-Millen transform
Source: PLoS One. 2025 Jun 17;20(6):e0321204. doi: 10.1371/journal.pone.0321204 (PMC12173420; doi:10.1371/journal.pone.0321204)
Supplement: S1 Code — This script executes the primary analysis, running the three models and applying profitability, error, training, and testing analyses. (PDF) [file pone.0321204.s001.pdf]

```
close all
clear
clc
%% Load fetched data and restrict attention to stocks without entry/
exit during the interval

load d1
s  (cellfun(@(x)isempty(x  ),d))=[];
d  (cellfun(@(x)isempty(x  ),d))=[];
% hist(cellfun(@(x)size(x,1),d))
% [C,ia,ic] = unique(cellfun(@(x)size(x,1),d));
% a_counts = accumarray(ic,1);
% value_counts = [C, a_counts]
N=                ...
max(cellfun(@(x)   size(x,1),d)   );
d=d(cellfun(@(x)   size(x,1),d)==N);

%% Plot the panel and convert to array

figure
K=              length(d)  ;
D=          d{1}.Date;
X=    zeros(N,K );
for i=      1:K
    semilogy(d{i}.Date,d{i}.Close);hold on
             X(: , i )=d{i}.Close ;
        disp(  i)
end
set  (   gcf,'PaperOrientation','landscape')
print('-dpng','/Users/muhammadalkhudaydi/Documents/Report_research/
fig/ fig10.png')
print('-dpdf','/Users/muhammadalkhudaydi/Documents/Report_research/
fig/ fig10.pdf')
print('fig10', '-dpng');close
%% Linear dimension reduction via PCA, VAR(1) estimation and
forecasting and AutoML

 % Compute log-returns
X=                ...
log(X(2:end   ,:)./...
    X(1:end-1 ,:));
[                 ...
Z,~,~,~,          ...
S,~               ...
]=                ...
pca(X) ;
p=min(  find(S<S(1)/1e2,1),1e1);
Z=Z(:,1:p)    ;
W=X*Z;

 % Rolling (daily) linear VAR(1) dynamical system models and
expected returns one-step-ahead

L= 22;  % Lag length
```

```
V=      varm(p,1);
P=      cell(size(D(1:end-L  )));
E=P;
for i =1:length(P)
  P{i}=estimate(V,W(i:i+L-1,:)) ;
  E{i}=zeros(1,p);
  for j =       max(1,i-L+1):i
  E{i}=            ...
  E{i}+forecast(   ...
  P{i},1         ,W(i:i+L-1,:)) ;
  end
  E{i}=            ...
  E{i}/         min(  i,L  );
  disp(i)
end

% %% State-space model estimation
%
% w=    iddata(W)  ;
% w.TimeUnit='days';
% s=     ssest(w,1 :  1e1  );
%      compare(w,s)
%
 % AutoML for p (portfolio) targets given (once-lagged) W

Y=cell2mat(E);
m=cell      (1,p);
y=m;
q=0.8;  % Training percentage
I=   floor(size(Y,1) *q);
U=                W(1:I,:);
u=                W(  I                        ...
                   +1:                         ...
           length(E)   ,:);
MaxTimeMin=10;
T=              Y(    I                        ...
                   +1:                         ...
                    end,:);
parfor i=1:size(Y,2)
m{i}=fitrauto(U,Y(1:I,i),'Learners','all'     ,...
          'OptimizeHyperparameters','all'     ,...
'HyperparameterOptimizationOptions',struct(    ...
                        'Optimizer','bayesopt',...
                          'MaxTime',           ...
                        60*MaxTimeMin));
y{i}.m.tr          ...
    = predict(     ...
m{i},         U)   ;
y{i}.m.te          ...
    = predict(     ...
m{i},         u)   ;
end
figure(1) ; sgtitle('AutoML Training')
figure(2) ; sgtitle('AutoML Testing' )
```

```
for    i=1:size(Y,2)
figure(1) ; subplot(floor(sqrt(p)) ,           ...
                     ceil(     p   /           ...
                    floor(sqrt(p))),i)
         plot(  Y(1:I,i),                      ...
y{i}.m.tr ,'o')    ;f=gca;f.Children.MarkerFaceColor=f.Children.Colo
r;f.Children(1).MarkerFaceColor=f.Children(1).Color;xlim([min(f.XLim
(1),f.YLim(1)) max(f.XLim(2),f.YLim(2))]);ylim(f.XLim)
                                       title(strcat('PC','
',num2str(i)));xlabel('True');ylabel('Predicted')
figure(2) ; subplot(floor(sqrt(p)) ,           ...
                     ceil(     p   /           ...
                    floor(sqrt(p))),i)
         plot(  T( : ,i),                      ...
y{i}.m.te,'o')     ;f=gca;f.Children.MarkerFaceColor=f.Children.Colo
r;f.Children(1).MarkerFaceColor=f.Children(1).Color;xlim([min(f.XLim
(1),f.YLim(1)) max(f.XLim(2),f.YLim(2))]);ylim(f.XLim)
                                       title(strcat('PC','
',num2str(i)));xlabel('True');ylabel('Predicted')
end
set  (   gcf,'PaperOrientation','landscape')
print('fig11', '-dpng');close
set  (   gcf,'PaperOrientation','landscape')
print('fig12', '-dpng');close
%% Continuous wavelet transform and CNN

Q=      cell(      ...
           length(D(1:end-L)),p);
for i =1:length(P)
for j =1:       p
Q{i,j}=     cwt(  W(i:i+L-1,j)) ;
end
  disp(i)
end

layers=[
    imageInputLayer([size(Q{1,1}) 1])
    convolution2dLayer(3, 8,'Padding','same')
    batchNormalizationLayer
    reluLayer
    averagePooling2dLayer(2,'Stride',2)
    convolution2dLayer(3,16,'Padding','same')
    batchNormalizationLayer
    reluLayer
    averagePooling2dLayer(2,'Stride',2)
    convolution2dLayer(3,32,'Padding','same')
    batchNormalizationLayer
    reluLayer
    convolution2dLayer(3,32,'Padding','same')
    batchNormalizationLayer
    reluLayer
    dropoutLayer(0.2)
    fullyConnectedLayer(1)
    regressionLayer];
```

```
miniBatchSize       = 128;
validationFrequency = floor(I/miniBatchSize);
options             =
trainingOptions('sgdm',                        ...
                             'MiniBatchSize',
miniBatchSize,       ...
                                 'MaxEpochs',
30,                  ...
                          'InitialLearnRate',
1e-3,               ...

'LearnRateSchedule','piecewise',          ...
                       'LearnRateDropFactor',
0.1,                ...
                       'LearnRateDropPeriod',
20,                  ...
                                   'Shuffle','every-
epoch',        ...
                       'ValidationFrequency',
validationFrequency, ...
                                     'Plots','training-
progress',  ...
                                   'Verbose', false);

M=cell      (1,p);
figure(3) ; sgtitle(   'CNN Training')
figure(4) ; sgtitle(   'CNN Testing' )
for    i=1:size(Y,2)
M{i}=             ...
 trainNetwork(abs( ...
        stack(Q  (1:I,i)               ))      ...
               ,Y(1:I,i),layers,options);
y{i}.M.tr         ...
    = predict(     ...
M{i},         abs( ...
        stack(Q  (1:I,i)             )));
y{i}.M.te         ...
    = predict(     ...
M{i},         abs( ...
        stack(Q  (  I                          ...
                 +1:                           ...
                  end,i)             )));
figure(3) ; subplot(floor(sqrt(p)) ,           ...
                     ceil(     p   /           ...
                    floor(sqrt(p))),i); hold on
         plot(  Y(1:I,i),                      ...
y{i}.M.tr ,'o')    ;f=gca;f.Children   .MarkerFaceColor=f.Children
.Color;xlim([min(f.XLim(1),f.YLim(1))
max(f.XLim(2),f.YLim(2))]);ylim(f.XLim)
                                       title(strcat('PC','
',num2str(i)));xlabel('True');ylabel('Predicted')
% legend({'AutoML','CNN'})
figure(4) ; subplot(floor(sqrt(p)) ,           ...
```

```
                     ceil(     p   /           ...
                    floor(sqrt(p))),i); hold on
         plot(  T( : ,i),                      ...
y{i}.M.te,'o')     ;f=gca;f.Children   .MarkerFaceColor=f.Children
.Color;xlim([min(f.XLim(1),f.YLim(1))
max(f.XLim(2),f.YLim(2))]);ylim(f.XLim)
                                       title(strcat('PC','
',num2str(i)));xlabel('True');ylabel('Predicted')
% legend({'AutoML','CNN'})
end
set  (   gcf,'PaperOrientation','landscape')
print('fig13', '-dpng');close
set  (   gcf,'PaperOrientation','landscape')
print('fig14', '-dpng');close

%% Fourier-Mellin transform and LSTM

J=      cell(      ...
           length(D(1:end-L)),p                ...
                             -1);
for i =1:length(P)
for j =1:       p-1
J{i,j}=     FM (abs(                           ...
Q{i,j})        ,abs(                           ...
Q{i,p}));
end
  disp(i)
end

layrs =[
    sequenceInputLayer(size(J{1,1},2))
    lstmLayer(200,'OutputMode','sequence')
    dropoutLayer(0.2)
    fullyConnectedLayer(1)
%     softmaxLayer
    regressionLayer];

options            =
trainingOptions('adam',                      ...
                             'L2Regularization',
0.001,          ...
                             'MiniBatchSize',
100,               ...
                                 'MaxEpochs',
30,                 ...
                          'InitialLearnRate',
1e-2,             ...

'LearnRateSchedule','piecewise',          ...
                       'LearnRateDropPeriod',
3,                ...
                         'GradientThreshold',
1,                ...
                                     'Plots','training-
```

```
progress',  ...
                                   'Shuffle','every-
epoch',        ...
                                   'Verbose',
false,              ...
                     'DispatchInBackground',  true);

O=cell      (1,p   ...
              -1);
figure(5) ; sgtitle(  'LSTM Training')
figure(6) ; sgtitle(  'LSTM Testing' )
for    i=1:size(Y,2)-1
O{i}=              ...
 trainNetwork(     ...
 cell2mat    (J  (1:I,i)             )'         ...
               ,Y(1:I,i)',layrs,options) ;
y{i}.O.tr          ...
    = predict(     ...
O{i},              ...
 cell2mat    (J  (1:I,i)             )')';
y{i}.O.te          ...
    = predict(     ...
O{i},              ...
 cell2mat    (J  (  I                          ...
                 +1:                           ...
                  end,i)             )')';
figure(5) ; subplot(floor(sqrt(p)) ,           ...
                     ceil(     p   /           ...
                    floor(sqrt(p))),i); hold on
         plot(  Y(1:I,i),                      ...
y{i}.O.tr ,'o')    ;f=gca;f.Children   .MarkerFaceColor=f.Children
.Color;xlim([min(f.XLim(1),f.YLim(1))
max(f.XLim(2),f.YLim(2))]);ylim(f.XLim)
                                       title(strcat('PC','
',num2str(i)));xlabel('True');ylabel('Predicted')
% legend({'AutoML','CNN'})
figure(6) ; subplot(floor(sqrt(p)) ,           ...
                     ceil(     p   /           ...
                    floor(sqrt(p))),i); hold on
         plot(  T( : ,i),                      ...
y{i}.O.te,'o')     ;f=gca;f.Children   .MarkerFaceColor=f.Children
.Color;xlim([min(f.XLim(1),f.YLim(1))
max(f.XLim(2),f.YLim(2))]);ylim(f.XLim)
                                       title(strcat('PC','
',num2str(i)));xlabel('True');ylabel('Predicted')
% legend({'AutoML','CNN'})
end
set  (   gcf,'PaperOrientation','landscape')
print('fig15', '-dpng');close
set  (   gcf,'PaperOrientation','landscape')
print('fig16', '-dpng');close
%% Profitability comparison

R=zeros(  length(E),3);
```

```
[   ~,  ...
R(1:I,1)...
]=  max(cell2mat(cellfun(@(x)x.m.tr,y,'UniformOutput',false)),[],2);
[   ~,  ...
R(1:I,2)...
]=  max(cell2mat(cellfun(@(x)x.M.tr,y,'UniformOutput',false)),[],2);
[   ~,  ...
R(1:I,3)...
]=  max(cell2mat(cellfun(@(x)x.O.tr,y(1:end-1) ...
                                    ,'UniformOutput',false)),[],2);
[   ~,  ...
R(  I   ...
 +1:end ...
     ,1)...
]=  max(cell2mat(cellfun(@(x)x.m.te,y,'UniformOutput',false)),[],2);
[   ~,  ...
R(  I   ...
 +1:end ...
     ,2)...
]=  max(cell2mat(cellfun(@(x)x.M.te,y,'UniformOutput',false)),[],2);
[   ~,  ...
R(  I   ...
 +1:end ...
     ,3)...
]=  max(cell2mat(cellfun(@(x)x.O.te,y(1:end-1) ...
                                    ,'UniformOutput',false)),[],2);
r=zeros(    size(R)  );
for i=1:  length(E)
r  (i,:)...
 =W(      length(D)...
 -        length(E)...
 -1+i,  ...
  R(i,:));
end
   plot(  cumsum(r));xticks([1 I size(r,1)]);xticklabels({'April
2013','Testing','April 2023'});hold on;grid on;f=gca;
   plot(I*  ones(1e+2
,1),linspace(f.YLim(1),f.YLim(2)),'k--');title('Cumulative (log)
returns');legend({'VAR(1)-AutoML','CWT-CNN','FM-LSTM'})
   print('fig17', '-dpng');
 %% save all workspace variables
filename = "report_matlb_1.mat";
save(filename)
%% Plot cumulative sum of r with a larger line width
plot(cumsum(r), 'LineWidth', 2);

% Add the x-axis ticks and labels
xticks([1 I size(r,1)]);
xticklabels({'April 2013','Testing','April 2023'});

% Label the x-axis
xlabel('Time');

% Label the y-axis
```

```
ylabel('Cumulative (log) returns');

% Hold on to the current plot
hold on;

% Turn the grid on
grid on;

% Get the handle to the current axes
f = gca;

% Add a vertical dashed line at the index I
plot(I * ones(1e+2 ,1), linspace(f.YLim(1),f.YLim(2)), 'k--',
'LineWidth', 2);

% Add a title to the plot
title('Path for each Investment Stratigey ');

% Add a legend to the plot with location set to 'best'
legend({'VAR(1)-AutoML','CWT-CNN','FM-LSTM'}, 'Location', 'best');
print('fig18', '-dpng');
%% Assuming Y is a matrix with training and test data combined
% Split Y into train and test based on your indices
Y_train = Y(1:1998, :); % 1998x10 for training
Y_test = Y(1999:end, :); % 500x10 for testing

% Assuming 'y' is your 1x10 cell array

% Pre-allocate matrices to store tr and te values
m_tr = zeros(1998, 10);
m_te = zeros(500, 10);
M_tr = zeros(1998, 10);
M_te = zeros(500, 10);
O_tr = zeros(1998, 9);  % Changed: O_tr now has 9 columns
O_te = zeros(500, 9);  % Changed: O_te now has 9 columns

% Loop through each cell
for i = 1:10
  % Access each struct within the cell
  current_struct = y{i};

  % Extract and store tr and te values for 'm'
  m_tr(:, i) = current_struct.m.tr;
  m_te(:, i) = current_struct.m.te;

  % Extract and store tr and te values for 'M'
  M_tr(:, i) = current_struct.M.tr;
  M_te(:, i) = current_struct.M.te;

  % Extract and store tr and te values for 'O' if it exists
  if i <= 9  % Check if 'O' exists in this struct
    O_tr(:, i) = current_struct.O.tr;
    O_te(:, i) = current_struct.O.te;
  end
```

```
end

% Now you have six matrices:
% - m_tr: 1998x10 double, containing all 'tr' values for 'm'
% - m_te: 500x10 double, containing all 'te' values for 'm'
% - M_tr: 1998x10 double, containing all 'tr' values for 'M'
% - M_te: 500x10 double, containing all 'te' values for 'M'
% - O_tr: 1998x9 double, containing all 'tr' values for 'O'
% - O_te: 500x9 double, containing all 'te' values for 'O'

%% Assuming 'tr' is your 1998x1 time series of true values
% Assuming 'prediction' is your 1998x1 time series of predictions

% Calculate Mean Absolute Error (MAE)
MAE = mean(abs(O_tr(:,1) - Y_train));

% Calculate Root Mean Squared Error (RMSE)
%% Calculate errors
errors = O_tr(:,2) - Y_train(:,3);

% Example for daily data over 1998 points
t = 1:1998; % or linspace(1, 1998, 1998)

% Plot the time series, predictions, and errors
figure;
hold on;

% Plot true values
p1 = plot(t, Y_train, 'g-', 'LineWidth', 2); % Green for true values

% Plot predictions
p2 = plot(t, O_tr(:,1), 'm--', 'LineWidth', 2); % Magenta for
predictions

% Plot errors (optional)
p3 = plot(t, errors, 'k:', 'LineWidth', 1); % Black for errors

% Add labels and title
xlabel('Time');
ylabel('Value');
title('Time Series, Predictions, and Errors');

% Add legend with handles
legend([p1(1), p2(1), p3(1)], {'True Values', 'Predictions',
'Errors'});

grid on;
hold off;
%% Error_analysis
[trainMetrics, testMetrics, results] =
calculateMetrics_csv_1(Y_train, Y_test, m_tr, M_tr, m_te, M_te,...
    'train_results_M.csv', 'test_results_M.csv',
'train_results_m.csv', 'test_results_m.csv');
[trainMetrics_O, testMetrics_O, results_1] =
```

```
calculateMetrics_csv_OO(Y_train, Y_test, O_tr,  O_te,...
    'train_results_O.csv', 'test_results_O.csv');
%% save again
filename = "test1.mat";
save(filename)
%%
% Get all variable names from the workspace
% Get all variable names from the workspace
vars = who;

% Number of variables
numVars = length(vars);

% Number of files
numFiles = 4;

% Variables per file
varsPerFile = ceil(numVars / numFiles);

for i = 1:numFiles
    % Calculate the start and end indices for the current file
    startIndex = (i - 1) * varsPerFile + 1;
    endIndex = min(i * varsPerFile, numVars);

    % Create the filename
    filename = sprintf('datafile_%d.mat', i);

     % Initialize a cell array to store variable names for this file
    variablesToSave = {};

    % Select three variables (or fewer if there are not enough left)
    for j = startIndex:min(endIndex, startIndex+2)
        if j <= numVars % Check if we are within the bounds of the
available variables
          variablesToSave{end+1} = vars{j};
        end
    end

    % Save the selected variables to the MAT file
    save(filename, variablesToSave{:});
end
```
