# Supplementary material for: Investigating the dynamics and uncertainties in portfolio optimization using the Fourier-Millen transform
Source: PLoS One. 2025 Jun 17;20(6):e0321204. doi: 10.1371/journal.pone.0321204 (PMC12173420; doi:10.1371/journal.pone.0321204)
Supplement: S2 Code — Helper function to stack data into a 2D vector using feature extraction for CNN and LSTM. (PDF) [file pone.0321204.s002.pdf]

```
function X=stack(Q)
         X=zeros( [   ...
            size(Q{1})...
        1 length(Q) ]) ;
 for i =1:length(Q)
         X      (:,:, ...
   1,i)=        Q{i}  ;
  end
end
```
