# Supplementary material for: Investigating the dynamics and uncertainties in portfolio optimization using the Fourier-Millen transform
Source: PLoS One. 2025 Jun 17;20(6):e0321204. doi: 10.1371/journal.pone.0321204 (PMC12173420; doi:10.1371/journal.pone.0321204)
Supplement: S3 Code — Applies the Fourier-Millen transform to extract geometric features. (PDF) [file pone.0321204.s003.pdf]

```
function params = FM(I1,I2)%im1, im2)
% global scale
% %% Loading images
% I1 = imresize(imread(im1), scale);
% I2 = imresize(imread(im2), scale);
% Convert both to FFT, centering on zero frequency component
SizeX = size(I1, 1);
SizeY = size(I1, 2);
FA = fftshift(fft2(I1));
FB = fftshift(fft2(I2));
% Output (FA, FB)
%%
--------------------------------------------------------------------
-
% Convolve the magnitude of the FFT with a high pass filter)
IA = hipass_filter(size(I1, 1),size(I1,2)).*abs(FA);
IB = hipass_filter(size(I2, 1),size(I2,2)).*abs(FB);
% Transform the high passed FFT phase to Log Polar space
L1 = transform_Image(IA, SizeX, SizeY, SizeX, SizeY, ...
 'nearest', size(IA) / 2, 'valid');
L2 = transform_Image(IB, SizeX, SizeY, SizeX, SizeY, ...
 'nearest', size(IB) / 2, 'valid');
% Convert log polar magnitude spectrum to FFT
THETA_F1 = fft2(L1);
THETA_F2 = fft2(L2);
% Compute cross power spectrum of F1 and F2
a1 = angle(THETA_F1);
a2 = angle(THETA_F2);
THETA_CROSS = exp(1i * (a1 - a2));
THETA_PHASE = real(ifft2(THETA_CROSS));
% Find the peak of the phase correlation
THETA_SORTED = sort(THETA_PHASE(:)); % TODO speed-up, we surely
don't need to sort
SI = length(THETA_SORTED):-1:(length(THETA_SORTED));
[THETA_X, THETA_Y] = find(THETA_PHASE == THETA_SORTED(SI));
% Compute angle of rotation
DPP = 360 / size(THETA_PHASE, 2);
Theta = DPP * (THETA_Y - 1);
% Output (Theta)
%%
--------------------------------------------------------------------
-
% Rotate image back by theta and theta + 180
R1 = imrotate(I2, -Theta, 'nearest', 'crop');
R2 = imrotate(I2,-(Theta + 180), 'nearest', 'crop');
% Output (R1, R2)
%%
--------------------------------------------------------------------
-
% Take FFT of R1
R1_F2 = fftshift(fft2(R1));
% Compute cross power spectrum of R1_F2 and F2
a1 = angle(FA);
a2 = angle(R1_F2);
```

```
R1_F2_CROSS = exp(1i * (a1 - a2));
R1_F2_PHASE = real(ifft2(R1_F2_CROSS));
% Output (R1_F2_PHASE)
%%
--------------------------------------------------------------------
-
% Take FFT of R2
R2_F2 = fftshift(fft2(R2));
% Compute cross power spectrum of R2_F2 and F2
a1 = angle(FA);
a2 = angle(R2_F2);
R2_F2_CROSS = exp(1i * (a1 - a2));
R2_F2_PHASE = real(ifft2(R2_F2_CROSS));
% Output (R2_F2_PHASE)
%%
--------------------------------------------------------------------
-
% Decide whether to flip 180 or -180 depending on which was the
closest
MAX_R1_F2 = max(max(R1_F2_PHASE));
MAX_R2_F2 = max(max(R2_F2_PHASE));
if (MAX_R1_F2 > MAX_R2_F2)
 [y, x] = find(R1_F2_PHASE == max(max(R1_F2_PHASE)));
 R = R1;
else
 [y, x] = find(R2_F2_PHASE == max(max(R2_F2_PHASE)));
 if (Theta < 180)
 Theta = Theta + 180;
 else
 Theta = Theta - 180;
 end
 R = R2;
end
% Output (R, x, y)
%%
--------------------------------------------------------------------
-
% Ensure correct translation by taking from correct edge
Tx = x - 1;
Ty = y - 1;
if (x > (size(I1, 1) / 2))
 Tx = Tx - size(I1, 1);
end
if (y > (size(I1, 2) / 2))
 Ty = Ty - size(I1, 2);
end
% Output (Sx, Sy)
%%
--------------------------------------------------------------------
-
% FOLLOWING CODE TAKEN DIRECTLY FROM fm_gui_v2
% Combine original and registered images
input2_rectified = R; move_ht = Ty; move_wd = Tx;
params = [MAX_R1_F2, MAX_R2_F2, move_ht, move_wd, Theta];
```

end
