# Supplementary material for: Investigating the dynamics and uncertainties in portfolio optimization using the Fourier-Millen transform
Source: PLoS One. 2025 Jun 17;20(6):e0321204. doi: 10.1371/journal.pone.0321204 (PMC12173420; doi:10.1371/journal.pone.0321204)
Supplement: S4 Code — High-pass filter function used within FM.m. (PDF) [file pone.0321204.s004.pdf]

```
%%%%% Important function. %%%%%
% Returns high-pass filter
function H = hipass_filter(ht,wd)
% hi-pass filter function
% ...designed for use with Fourier-Mellin stuff
res_ht = 1 / (ht-1);
res_wd = 1 / (wd-1);
eta = cos(pi*(-0.5:res_ht:0.5));
neta = cos(pi*(-0.5:res_wd:0.5));
X = eta'*neta;
H=(1.0-X).*(2.0-X);
end
```
