# Supplementary material for: Investigating the dynamics and uncertainties in portfolio optimization using the Fourier-Millen transform
Source: PLoS One. 2025 Jun 17;20(6):e0321204. doi: 10.1371/journal.pone.0321204 (PMC12173420; doi:10.1371/journal.pone.0321204)
Supplement: S5 Code — Computes MAE and RMSE and plots them as time series. (PDF) [file pone.0321204.s005.pdf]

```
%%
% Assuming Y is a matrix with training and test data combined
% Split Y into train and test based on your indices
Y_train = Y(1:1998, :); % 1998x10 for training
Y_test = Y(1999:end, :); % 500x10 for testing

% Assuming 'y' is your 1x10 cell array

% Pre-allocate matrices to store tr and te values
m_tr = zeros(1998, 10);
m_te = zeros(500, 10);
M_tr = zeros(1998, 10);
M_te = zeros(500, 10);
O_tr = zeros(1998, 9);  % Changed: O_tr now has 9 columns
O_te = zeros(500, 9);  % Changed: O_te now has 9 columns

% Loop through each cell
for i = 1:10
  % Access each struct within the cell
  current_struct = y{i};

  % Extract and store tr and te values for 'm'
  m_tr(:, i) = current_struct.m.tr;
  m_te(:, i) = current_struct.m.te;

  % Extract and store tr and te values for 'M'
  M_tr(:, i) = current_struct.M.tr;
  M_te(:, i) = current_struct.M.te;

  % Extract and store tr and te values for 'O' if it exists
  if i <= 9  % Check if 'O' exists in this struct
    O_tr(:, i) = current_struct.O.tr;
    O_te(:, i) = current_struct.O.te;
  end
end

% Now you have six matrices:
% - m_tr: 1998x10 double, containing all 'tr' values for 'm'
% - m_te: 500x10 double, containing all 'te' values for 'm'
% - M_tr: 1998x10 double, containing all 'tr' values for 'M'
% - M_te: 500x10 double, containing all 'te' values for 'M'
% - O_tr: 1998x9 double, containing all 'tr' values for 'O'
% - O_te: 500x9 double, containing all 'te' values for 'O'

%%
% Assuming 'tr' is your 1998x1 time series of true values
% Assuming 'prediction' is your 1998x1 time series of predictions

% Calculate Mean Absolute Error (MAE)
MAE = mean(abs(O_tr(:,2) - Y_train(:,3)));

% Calculate Root Mean Squared Error (RMSE)
%%
% Calculate errors
```

```
errors = M_tr(:,3) - Y_train(:,3);

% Example for daily data over 1998 points
t = 1:1998; % or linspace(1, 1998, 1998)

% Plot the time series, predictions, and errors
figure;
hold on;

% Plot true values
p1 = plot(t,  Y_train(:,3), 'g-', 'LineWidth', 2); % Green for true
values

% Plot predictions
p2 = plot(t, m_tr(:,1), 'm--', 'LineWidth', 2); % Magenta for
predictions

% Plot errors (optional)
p3 = plot(t, errors, 'k:', 'LineWidth', 1); % Black for errors

% Add labels and title
xlabel('Time');
ylabel('Value');
title('Time Series, Predictions, and Errors');

% Add legend with handles
legend([p1(1), p2(1), p3(1)], {'True Values', 'Predictions',
'Errors'});

grid on;
hold off;

%%
% Calculate errors
errors = O_tr(:,2) - Y_train(:,3);

% Example for daily data over 1998 points
t = 1:1998; % or linspace(1, 1998, 1998)

% Plot the time series, predictions, and errors
figure;

% Plot true values
subplot(3,1,1);
plot(t, Y_train, 'g-', 'LineWidth', 2); % Green for true values
xlabel('Time');
ylabel('True Values');
title('True Values');
grid on;

% Plot predictions
subplot(3,1,2);
plot(t, O_tr(:,1), 'm--', 'LineWidth', 2); % Magenta for predictions
xlabel('Time');
```

```
ylabel('Predictions');
title('Predictions');
grid on;

% Plot errors
subplot(3,1,3);
plot(t, errors, 'k:', 'LineWidth', 1); % Black for errors
xlabel('Time');
ylabel('Errors');
title('Errors');
grid on;

% Overall Title
sgtitle('Time Series, Predictions, and Errors');
```
