# Supplementary material for: Investigating the dynamics and uncertainties in portfolio optimization using the Fourier-Millen transform
Source: PLoS One. 2025 Jun 17;20(6):e0321204. doi: 10.1371/journal.pone.0321204 (PMC12173420; doi:10.1371/journal.pone.0321204)
Supplement: S6 Code — Applies MAE and RMSE on VAR(1)-AutoML and CWT-CNN, storing results as CSV files. (PDF) [file pone.0321204.s006.pdf]

```
function [trainMetrics, testMetrics, results] =
calculateMetrics_csv_1(Y_train, Y_test, m_tr, M_tr, m_te, M_te,
filenameTrainM, filenameTestM, filenameTrainm, filenameTestm)
    format long g
    % Initialize matrices to store metrics for each model and
dataset
    numSeries = size(Y_train, 2);
    trainMetrics = zeros(4, 2, numSeries); % 4 metrics x 2 methods x
numSeries
    testMetrics = zeros(4, 2, numSeries);

    % Loop over each time series
    for i = 1:numSeries
        % Training metrics
        trainMetrics(:, 1, i) = computeMetrics(Y_train(:, i),
m_tr(:, i));
        trainMetrics(:, 2, i) = computeMetrics(Y_train(:, i),
M_tr(:, i));

        % Testing metrics
        testMetrics(:, 1, i) = computeMetrics(Y_test(:, i), m_te(:,
i));
        testMetrics(:, 2, i) = computeMetrics(Y_test(:, i), M_te(:,
i));
    end

    % Extract MAE and MSE for each method
    trainMAEm = reshape(trainMetrics(1:2, 1, :), 2, numSeries)';
    testMAEm = reshape(testMetrics(1:2, 1, :), 2, numSeries)';

    trainMAEM = reshape(trainMetrics(1:2, 2, :), 2, numSeries)';
    testMAEM = reshape(testMetrics(1:2, 2, :), 2, numSeries)';

    % Create tables with headers
    trainResultsm = array2table(trainMAEm, 'VariableNames', {'MAE',
'MSE'});
    testResultsm = array2table(testMAEm, 'VariableNames', {'MAE',
'MSE'});
    trainResultsM = array2table(trainMAEM, 'VariableNames', {'MAE',
'MSE'});
    testResultsM = array2table(testMAEM, 'VariableNames', {'MAE',
'MSE'});

    % Write to CSV files
    writetable(trainResultsm, filenameTrainm); % 'm' method training
    writetable(testResultsm, filenameTestm); % 'm' method testing
    writetable(trainResultsM, filenameTrainM); % 'M' method training
    writetable(testResultsM, filenameTestM); % 'M' method testing

    % Combine results for output
    results.trainM = trainResultsM;
    results.testM = testResultsM;
    results.trainm = trainResultsm;
    results.testm = testResultsm;
```

```
end

function metrics = computeMetrics(yTrue, yPred)
    % Calculate MAE
    mae = mean(abs(yTrue - yPred));

    % Calculate MSE
    mse = mean((yTrue - yPred).^2);

    % Calculate R^2
    ssRes = sum((yTrue - yPred).^2);
    ssTot = sum((yTrue - mean(yTrue)).^2);
    r2 = 1 - (ssRes / ssTot);

    % Calculate MAPE
    mape = mean(abs((yTrue - yPred) ./ yTrue)) * 100;

    % Store metrics
    metrics = [mae; mse; r2; mape];
end
```
