# Supplementary material for: Investigating the dynamics and uncertainties in portfolio optimization using the Fourier-Millen transform
Source: PLoS One. 2025 Jun 17;20(6):e0321204. doi: 10.1371/journal.pone.0321204 (PMC12173420; doi:10.1371/journal.pone.0321204)
Supplement: S7 Code — Applies MAE and RMSE on FM-LSTM. (PDF) [file pone.0321204.s007.pdf]

```
function [trainMetrics, testMetrics, results_1] =
calculateMetrics_csv_OO(Y_train, Y_test, O_tr,  O_te,
filenameTrainO, filenameTestO)
    format long g
    % Initialize matrices to store metrics for each model and
dataset
    numSeries = size(Y_train, 2)-1;
    trainMetrics = zeros(4, 1, numSeries); % 4 metrics x 2 methods x
numSeries
    testMetrics = zeros(4, 1, numSeries);

    % Loop over each time series
    for i = 1:numSeries
        % Training metrics
        trainMetrics(:, 1, i) = computeMetrics(Y_train(:, i),
O_tr(:, i));


        % Testing metrics
        testMetrics(:, 1, i) = computeMetrics(Y_test(:, i), O_te(:,
i));

    end

    % Extract MAE and MSE for each method
    trainMAEO = reshape(trainMetrics(1:2, 1, :), 2, numSeries)';
    testMAEO = reshape(testMetrics(1:2, 1, :), 2, numSeries)';



    % Create tables with headers
    trainResultsO = array2table(trainMAEO, 'VariableNames', {'MAE',
'MSE'});
    testResultsO = array2table(testMAEO, 'VariableNames', {'MAE',
'MSE'});


    % Write to CSV files
    writetable(trainResultsO, filenameTrainO); % 'm' method training
    writetable(testResultsO, filenameTestO); % 'm' method testing


    % Combine results for output
    results_1.trainO = trainResultsO;
    results_1.testO = testResultsO;

end

function metrics = computeMetrics(yTrue, yPred)
    % Calculate MAE
    mae = mean(abs(yTrue - yPred));

    % Calculate MSE
    mse = mean((yTrue - yPred).^2);
```

```
    % Calculate R^2
    ssRes = sum((yTrue - yPred).^2);
    ssTot = sum((yTrue - mean(yTrue)).^2);
    r2 = 1 - (ssRes / ssTot);

    % Calculate MAPE
    mape = mean(abs((yTrue - yPred) ./ yTrue)) * 100;

    % Store metrics
    metrics = [mae; mse; r2; mape];
end
```
