# Supplementary material for: Investigating the dynamics and uncertainties in portfolio optimization using the Fourier-Millen transform
Source: PLoS One. 2025 Jun 17;20(6):e0321204. doi: 10.1371/journal.pone.0321204 (PMC12173420; doi:10.1371/journal.pone.0321204)
Supplement: S8 Code — Transforms 2D images to obtain geometric features. (PDF) [file pone.0321204.s008.pdf]

```
function [r,g,b] =
transform_Image(A,Ar,Ac,Nrho,Ntheta,Method,Center,Shape)
% Inputs:   A       the input image
%           Nrho    the desired number of rows of transformed image
%           Ntheta  the desired number of columns of transformed
image
%           Method  interpolation method (nearest,bilinear,bicubic)
%           Center  origin of input image
%           Shape   output size (full,valid)
%           Class   storage class of A

global rho;

theta = linspace(0,2*pi,Ntheta+1); theta(end) = [];

switch Shape
case 'full'
    corners = [1 1;Ar 1;Ar Ac;1 Ac];
    d = max(sqrt(sum((repmat(Center(:)',4,1)-corners).^2,2)));
case 'valid'
    d = min([Ac-Center(1) Center(1)-1 Ar-Center(2) Center(2)-1]);
end
minScale = 1;
rho = logspace(log10(minScale),log10(d),Nrho)';  % default 'base 10'
logspace - play with d to change the scale of the log axis

% convert polar coordinates to cartesian coordinates and center
xx = rho*cos(theta) + Center(1);
yy = rho*sin(theta) + Center(2);

if nargout==3
  if strcmp(Method,'nearest'), % Nearest neighbor interpolation
    r=interp2(A(:,:,1),xx,yy,'nearest');
    g=interp2(A(:,:,2),xx,yy,'nearest');
    b=interp2(A(:,:,3),xx,yy,'nearest');
  elseif strcmp(Method,'bilinear'), % Linear interpolation
    r=interp2(A(:,:,1),xx,yy,'linear');
    g=interp2(A(:,:,2),xx,yy,'linear');
    b=interp2(A(:,:,3),xx,yy,'linear');
  elseif strcmp(Method,'bicubic'), % Cubic interpolation
    r=interp2(A(:,:,1),xx,yy,'cubic');
    g=interp2(A(:,:,2),xx,yy,'cubic');
    b=interp2(A(:,:,3),xx,yy,'cubic');
  else
    error(['Unknown interpolation method: ',method]);
  end
  % any pixels outside , pad with black
  mask= (xx>Ac) | (xx<1) | (yy>Ar) | (yy<1);
  r(mask)=NaN;
  g(mask)=NaN;
  b(mask)=NaN;
else
  if strcmp(Method,'nearest'), % Nearest neighbor interpolation
    r=interp2(A,xx,yy,'nearest');
```

```
  elseif strcmp(Method,'bilinear'), % Linear interpolation
    r=interp2(A,xx,yy,'linear');
  elseif strcmp(Method,'bicubic'), % Cubic interpolation
    r=interp2(A,xx,yy,'cubic');
  else
    error(['Unknown interpolation method: ',method]);
  end
  % any pixels outside warp, pad with black
  mask= (xx>Ac) | (xx<1) | (yy>Ar) | (yy<1);
  r(mask)=NaN;
end

end
```
