# Supplementary material for: Investigating the dynamics and uncertainties in portfolio optimization using the Fourier-Millen transform
Source: PLoS One. 2025 Jun 17;20(6):e0321204. doi: 10.1371/journal.pone.0321204 (PMC12173420; doi:10.1371/journal.pone.0321204)
Supplement: S9 Code — Implements statistical analysis. (PDF) [file pone.0321204.s009.pdf]

```
close all
clear
clc
%% Load fetched data and restrict attention to stocks without entry/
exit during the interval

load d1
%s  (cellfun(@(x)isempty(x  ),d))=[];
d  (cellfun(@(x)isempty(x  ),d))=[];
% hist(cellfun(@(x)size(x,1),d))
% [C,ia,ic] = unique(cellfun(@(x)size(x,1),d));
% a_counts = accumarray(ic,1);
% value_counts = [C, a_counts]
N=                ...
max(cellfun(@(x)   size(x,1),d)   );
d=d(cellfun(@(x)   size(x,1),d)==N);

%% Plot the panel and convert to array

figure
K=              length(d)  ;
D=          d{1}.Date;
X=    zeros(N,K );
for i=      1:K
    semilogy(d{i}.Date,d{i}.Close);hold on
            X(: , i )=d{i}.Close ;
        disp(  i)
end
set  (   gcf,'PaperOrientation','landscape')
print(strcat('/Path',num2str(i)),'-dpdf','-fillpage','-r0')

print('-dpng','/Path/ time_series.png')
%%
% Calculate mean
meanReturns = mean(X);

% Calculate median
medianReturns = median(X);

% Calculate standard deviation
stdReturns = std(X);

% Calculate skewness
skewnessReturns = skewness(X);

% Calculate kurtosis
kurtosisReturns = kurtosis(X);
n = numel(meanReturns); % get the number of stocks based on your
data

summaryTable = array2table([meanReturns; medianReturns; stdReturns;
skewnessReturns; kurtosisReturns]', ...
    'VariableNames', {'Mean', 'Median', 'StdDev', 'Skewness',
'Kurtosis'}, ...
```

```
    'RowNames', arrayfun(@(x) sprintf('Stock%d', x), 1:n,
'UniformOutput', false));

%%
% Calculate statistics
meanReturns = mean(X);
medianReturns = median(X);
stdReturns = std(X);
skewnessReturns = skewness(X);
kurtosisReturns = kurtosis(X);

% Create table
summaryTable = table(min(meanReturns), max(meanReturns),
mean(meanReturns), median(meanReturns), std(meanReturns),
'VariableNames', {'Min', 'Max', 'Mean', 'Median', 'StdDev'});
summaryTable.Properties.RowNames = {'Mean Return'};

summaryTable = [summaryTable; table(min(medianReturns),
max(medianReturns), mean(medianReturns), median(medianReturns),
std(medianReturns), 'VariableNames', {'Min', 'Max', 'Mean',
'Median', 'StdDev'})];
summaryTable.Properties.RowNames{end} = 'Median Return';

summaryTable = [summaryTable; table(min(stdReturns),
max(stdReturns), mean(stdReturns), median(stdReturns),
std(stdReturns), 'VariableNames', {'Min', 'Max', 'Mean', 'Median',
'StdDev'})];
summaryTable.Properties.RowNames{end} = 'Std Dev';

summaryTable = [summaryTable; table(min(skewnessReturns),
max(skewnessReturns), mean(skewnessReturns),
median(skewnessReturns), std(skewnessReturns), 'VariableNames',
{'Min', 'Max', 'Mean', 'Median', 'StdDev'})];
summaryTable.Properties.RowNames{end} = 'Skewness';

summaryTable = [summaryTable; table(min(kurtosisReturns),
max(kurtosisReturns), mean(kurtosisReturns),
median(kurtosisReturns), std(kurtosisReturns), 'VariableNames',
{'Min', 'Max', 'Mean', 'Median', 'StdDev'})];
summaryTable.Properties.RowNames{end} = 'Kurtosis';

% Display table
disp(summaryTable)
%%
% Write the summaryTable to a CSV file
writetable(summaryTable, 'summaryTable.csv');
%% Heat-map correlation
% Assuming 'returns' is a matrix where each column represents a
stock and
% each row represents a time period
% Assuming 'returns' is a matrix where each column represents a
stock and
% each row represents a time period
```

```
% Calculate correlation matrix
corrMatrix = corrcoef(X);

% Convert the correlation matrix to a dissimilarity matrix
dissimilarity = 1 - abs(corrMatrix);

% Perform hierarchical clustering
Z = linkage(dissimilarity, 'average');

% Create a dendrogram
figure
dendrogram(Z, 30); % Displays the last 30 merges
title('Hierarchical Clustering Dendrogram')
xlabel('Stock')
ylabel('Distance')
print('-dpng','/Path/fig/fig2.png')
%%
% Assuming 'returns' is your data matrix
%returns = rand(1421, 100); % Replace this with your actual data

% Compute the correlation matrix
corrMatrix = corrcoef(X);

% Compute the average correlation for each sample
avgCorr = mean(corrMatrix, 2);

% Define correlation groups based on the average correlation
% You can adjust the boundaries of the groups to suit your needs
lowThreshold = -0.8;
mediumThreshold = 0;
highThreshold = 0.2;
lowCorrGroup = find(avgCorr <= lowThreshold);
mediumCorrGroup = find(avgCorr > lowThreshold & avgCorr <=
mediumThreshold);
highCorrGroup = find(avgCorr > mediumThreshold & avgCorr <=
highThreshold);

% Generate the bar plot
figure;

hold on;
bar(lowCorrGroup, avgCorr(lowCorrGroup), 'r'); % Red for "Low"
bar(mediumCorrGroup, avgCorr(mediumCorrGroup), 'g'); % Green for
"Medium"
bar(highCorrGroup, avgCorr(highCorrGroup), 'b'); % Blue for "High"
hold off;

% Add labels
xlabel('Sample Index');
ylabel('Average Correlation');
title('Average Correlation of Samples Grouped by Correlation
Level');

% Add a legend
```

```
legend('Low', 'Medium', 'High');
print('-dpng','/Path/fig/fig4.png')

%% Linear dimension reduction via PCA, VAR(1) estimation and
forecasting and AutoML

 % Compute log-returns
X=                ...
log(X(2:end   ,:)./...
    X(1:end-1 ,:));
[                 ...
Z,score,latent,tsq,           ...
S,~               ...
]=                ...
pca(X) ;
p=min(  find(S<S(1)/1e2,1),1e1);
Z=Z(:,1:p)    ;
W=X*Z;
 % Rolling (daily) linear VAR(1) dynamical system models and
expected returns one-step-ahead

L= 22;  % Lag length
V=      varm(p,1);
P=      cell(size(D(1:end-L  )));
E=P;
for i =1:length(P)
  P{i}=estimate(V,W(i:i+L-1,:)) ;
  E{i}=zeros(1,p);
  for j =       max(1,i-L+1):i
  E{i}=             ...
  E{i}+forecast(    ...
  P{i},1         ,W(i:i+L-1,:)) ;
  end
  E{i}=             ...
  E{i}/         min(  i,L  );
  disp(i)
end
Q=      cell(      ...
           length(D(1:end-L)),p);
for i =1:length(P)
for j =1:       p
Q{i,j}=     cwt(  W(i:i+L-1,j)) ;
end
  disp(i)
end
%%
% Plot the first 10 principal components
% Plot the first 10 principal components
figure;
for i = 1:10
    subplot(5, 2, i);
    plot(W(:, i));
    xlabel('Time (Days)');
    ylabel(['PCA' num2str(i)]);
```

```
    grid on;
end
% Adjust the layout
%sgtitle('First 10 Principal Components of Stock Log Returns');
print('-dpng','/Path/fig/fig5.png')
%%
% Calculate the correlation matrix
corrMatrix = corrcoef(W);

% Plot the correlation heatmap
figure;
imagesc(corrMatrix);
colorbar;
colormap('parula');
%title('Correlation Heatmap of the Principal Components');
xticks(1:10);
yticks(1:10);
xlabel('Principal Component');
ylabel('Principal Component');
print('-dpng','/Path/fig/fig6.png')
%%
% Plot the correlation heatmap
figure;
imagesc(corrMatrix);
colorbar;
colormap('parula');
title('Correlation Heatmap of the Principal Components');
xticks(1:10);
yticks(1:10);
xlabel('Principal Component');
ylabel('Principal Component');

% Add correlation values to the heatmap
for i = 1:10
    for j = 1:10
        text(i, j, num2str(corrMatrix(j, i), '%0.2f'), ...
            'HorizontalAlignment', 'center', ...
            'Color', 'k');
    end
end
%%
% Assume PCs is your principal components matrix

% Calculate returns (change in value) for the principal components
%returns = diff(PCs)./PCs(1:end-1,:);

% Calculate cumulative returns
cumulativeReturns = cumprod(1 + W);

% Plot cumulative returns
figure;
hold on;
for i = 1:size(cumulativeReturns, 2)
    plot(cumulativeReturns(:, i));
```

```
end
hold off;
title('Cumulative Returns of Principal Components');
xlabel('Time');
ylabel('Cumulative Returns');
legend('PC1', 'PC2', 'PC3', 'PC4', 'PC5', 'PC6', 'PC7', 'PC8',
'PC9', 'PC10');
print('-dpdf','/Path/fig/fig7.pdf')
%%
% Assume PCs is your principal components matrix
% Assume weights is a 1x10 vector with the weights of each PC in the
portfolio

% Calculate weights based on variance explained
weights = Z(1:p)/sum(Z(1:p));

% Calculate portfolio log returns
portfolioLogReturns = W * weights';

% Calculate cumulative log return of the portfolio
cumulativePortfolioLogReturn = cumsum(portfolioLogReturns);

% Plot cumulative log return of the portfolio
figure;
plot(cumulativePortfolioLogReturn);
title('Cumulative Log Return of Portfolio');
xlabel('Time');
ylabel('Cumulative Log Return');
print('-dpdf','/Path/fig/fig8.pdf')
%%
figure;

% Asset 1
subplot(2,2,1)
[wt1, f1] = cwt(W(:,1));
pcolor(1:numel(W(:,1)),log2(f1),abs(wt1));
shading interp;
title('Wavelet transform of asset 1-PCA');
xlabel('Time Step');
ylabel('Scale');

% Asset 2
subplot(2,2,2)
[wt2, f2] = cwt(W(:,2));
pcolor(1:numel(W(:,2)),log2(f2),abs(wt2));
shading interp;
title('Wavelet transform of asset 2-PCA');
xlabel('Time Step');
ylabel('Scale');

% Asset 3
subplot(2,2,3)
[wt3, f3] = cwt(W(:,3));
pcolor(1:numel(W(:,3)),log2(f3),abs(wt3));
```

```
shading interp;
title('Wavelet transform of asset 3-PCA');
xlabel('Time Step');
ylabel('Scale');

% Asset 4
subplot(2,2,4)
[wt4, f4] = cwt(W(:,4));
pcolor(1:numel(W(:,4)),log2(f4),abs(wt4));
shading interp;
title('Wavelet transform of asset 4-PCA');
xlabel('Time Step');
ylabel('Scale');

% Save the figure as a PNG file
print('-dpdf','/Path/fig/fig9.pdf')
```
