# Supplementary material for: Investigating the dynamics and uncertainties in portfolio optimization using the Fourier-Millen transform
Source: PLoS One. 2025 Jun 17;20(6):e0321204. doi: 10.1371/journal.pone.0321204 (PMC12173420; doi:10.1371/journal.pone.0321204)
Supplement: S1 File — (PDF) [file pone.0321204.s010.pdf]

A description of the code usage and organization can be found below.

Here is an explanation on how to use the code to run the models that have been used in our research paper “Investigating the dynamics and uncertainties in portfolio optimization using the Fourier-Millen Transform.

This project contains the following MATLAB scripts:

1. main.m: This is the main code that runs the three models and apply analysis (profitability+ error+ training process + testing process) .
2. stack.m: This is a helper function that called in the main code to stack data to 2D vector using feature extraction approach to work with CNN and LSTM.
3. FM.m: This is a helper function that called in the main code to apply Fourier-Millen transform to extract geometric features.
4. hipass_filter.m: This is a helper function for FM.m that called in the main code to apply high-pass filter of 2-D vector.
5. error_analsis.m: This code applies MAE and RMSE and plot them as time series.
6. calculateMetrics_csv_1.m: This code applies MAE and RMSE on VAR(1)-AutoML (Donated as M in the main code) and CWT-CNN (Donated as m in the main code) and store them as csv file
7. calculateMetrics_csv_OO.m: This code applies MAE and RMSE on FM-LSTM (Donated as O in the main code).
8. transform_Image.m: This code transforms 2-D to obtain the geometric features.
9. staticstical_analysis.m: This code implements main statistical analysis.

FM.m, hipass_filter.m and transform_Image.m was taken from :

Robinson Laundon (2024). Fourier Mellin Image Registration (https://www.mathworks.com/matlabcentral/fileexchange/19731-fourier-mellin-image-registration), MATLAB Central File Exchange. Retrieved October 23, 2024.

In this project, we obtained our data by the help of Money.Net connection. In order to use this feature you must create an account. Here is the link for more details:

https://www.mathworks.com/help/datafeed/moneynet.html

However, we stored our data and research finding using the format *.mat as follows:

1. d1.mat: The data of 1421 stocks using MATLAB function of money.net site stored as MAT file.
2. reseach_report.mat: These all-main results obtained from three models (training and testing) to save time of running the code again.

How to run the code:

In order to run the model, you must put all these files in one folder (working directory) then, you run the main.m by MATLAB. Note, that you must have a licence to use MATLAB with the following packages:

Computer Vision Toolbox, Control System Toolbox, Curve Fitting Toolbox, Deep Learning Toolbox, DSP System Toolbox, Econometrics Toolbox, Financial Toolbox, Image Processing Toolbox, Instrument Control Toolbox, Optimization Toolbox, Parallel Computing Toolbox, Reinforcement Learning Toolbox, Signal Processing Toolbox, Simulink, Statistics and Machine Learning Toolbox, Symbolic Math Toolbox, Text Analytics Toolbox, and the Wavelet Toolbox.

To replicate our finding, you must upload report_matlab.mat which is saved as report_matlab.zip and plot the profitability plot. For other statistical results and plots you need to use staticstical_analysis.m on d1.mat.
