# Supplementary material for: Investigating the dynamics and uncertainties in portfolio optimization using the Fourier-Millen transform
Source: PLoS One. 2025 Jun 17;20(6):e0321204. doi: 10.1371/journal.pone.0321204 (PMC12173420; doi:10.1371/journal.pone.0321204)
Supplement: S2 File — (PDF) [file pone.0321204.s011.pdf]

**Variable**	**Description**	**Purpose/Computation**
**X**	Matrix of log-returns	`log(X(2:end, :) ./ X(1:end-1, :))`
**Z**	PCA scores matrix	Transformed data for dimensionality reduction
**S**	Singular values from PCA	Helps in selecting principal components
**p**	Number of principal components	Based on threshold of singular values
**W**	Data projected onto principal components	Input for subsequent models
**L**	Lag length for VAR(1) model	22
**V**	VAR(1) model specification object	-
**P**	Estimated VAR(1) models	Stores models for rolling windows
**E**	Expected one-step-ahead returns	Averaged forecasts from VAR models
**Y**	Matrix of expected returns	Derived from `E`
**m**, **y**	Placeholder arrays	For model outputs or further calculations
**q**	Training percentage	0.8
**I**	Index for splitting training and test data	-
**U**	Training data matrix	-
**u**	Test data matrix	-
**T**	Target matrix for test data	Expected returns

Table 1: Summary of Variables
